# Clinical ethics consultations: a scoping review of reported outcomes

**DOI:** 10.1186/s12910-022-00832-6

**Published:** 2022-09-27

**Authors:** Jennifer A. H. Bell, Marina Salis, Eryn Tong, Erica Nekolaichuk, Claudia Barned, Andria Bianchi, Daniel Z. Buchman, Kevin Rodrigues, Ruby R. Shanker, Ann M. Heesters

**Affiliations:** 1 Department of Clinical and Organizational Ethics, University Health Network, Toronto, ON Canada; 2 Department of Supportive Care Research, Princess Margaret Cancer Centre, University Health Network, Toronto, ON Canada; 3 The Institute for Education Research, University Health Network, Toronto, ON Canada; 4 Dalla Lana School of Public Health and Joint Centre for Bioethics, University of Toronto, Toronto, ON Canada; 5 KITE Research Institute, Toronto Rehabilitation, Toronto, ON Canada; 6 Centre for Addiction and Mental Health, Toronto, ON Canada; 7 Gerstein Science Information Centre, University of Toronto Libraries, University of Toronto, Toronto, ON Canada; 8 Pragmatic Health Ethics Research Unit, Institut de Recherches Cliniques de Montreal, Montreal, QC Canada; 9 Department of Psychiatry, University of Toronto, Toronto, ON Canada; 10 Department of Philosophy, University of Toronto, Toronto, ON Canada; 11 William Osler Health System, Brampton, ON Canada

**Keywords:** Clinical ethics consultation, Moral case deliberation, Scoping review, Outcomes, Effectiveness research

## Abstract

**Background:**

Clinical ethics consultations (CEC) can be complex interventions, involving multiple methods, stakeholders, and competing ethical values. Despite longstanding calls for rigorous evaluation in the field, progress has been limited. The Medical Research Council (MRC) proposed guidelines for evaluating the effectiveness of complex interventions. The evaluation of CEC may benefit from application of the MRC framework to advance the transparency and methodological rigor of this field. A first step is to understand the outcomes measured in evaluations of CEC in healthcare settings.

**Objective:**

The primary objective of this review was to identify and map the outcomes reported in primary studies of CEC. The secondary objective was to provide a comprehensive overview of CEC structures, processes, and roles to enhance understanding and to inform standardization.

**Methods:**

We searched electronic databases to identify primary studies of CEC involving patients, substitute decision-makers and/or family members, clinicians, healthcare staff and leaders. Outcomes were mapped across five conceptual domains as identified a priori based on our clinical ethics experience and preliminary literature searches and revised based on our emerging interpretation of the data. These domains included personal factors, process factors, clinical factors, quality, and resource factors.

**Results:**

Forty-eight studies were included in the review. Studies were highly heterogeneous and varied considerably regarding format and process of ethical intervention, credentials of interventionist, population of study, outcomes reported, and measures employed. In addition, few studies used validated measurement tools. The top three outcome domains that studies reported on were quality (n = 31), process factors (n = 23), and clinical factors (n = 19)**.** The majority of studies examined multiple outcome domains. All five outcome domains were multidimensional and included a variety of subthemes.

**Conclusions:**

This scoping review represents the initial phase of mapping the outcomes reported in primary studies of CEC and identifying gaps in the evidence. The confirmed lack of standardization represents a hindrance to the provision of high quality intervention and CEC scientific progress. Insights gained can inform the development of a core outcome set to standardize outcome measures in CEC evaluation research and enable scientifically rigorous efficacy trials of CEC.

## Introduction

Clinical ethics consultations (CEC) can be complex interventions involving multiple methods, stakeholders, and ethical principles or values, often in conflict. Despite longstanding calls for rigorous evaluation in the field of clinical ethics [1], progress continues to be limited. The Medical Research Council (MRC) proposed guidelines for evaluating the effectiveness of complex interventions [2]. Relevant stages to consider include the selection of appropriate study designs, identification of important outcomes, understanding processes, and the assessment of intervention fidelity. The evaluation of CEC may benefit from application of the MRC framework to advance the transparency and methodological rigour of this field [3]. A necessary first step is to understand the types of outcomes measured in primary studies of CEC in healthcare settings.

## Background

CEC is broadly understood as “a service provided by an individual consultant, team, or committee to address the ethical issues involved in a specific clinical case. Its central purpose is to improve the process and outcomes of patient care by helping to identify, analyze, and resolve ethical problems” [4]. This definition encompasses a variety of clinical ethics cases, from the relatively straightforward (e.g., clarifying the role of the substitute decision maker) to more complex (e.g., mediating entrenched values-disagreement) and service models, including moral case deliberation, whereby the clinical ethicist is a well-trained facilitator with ethics knowledge. Other models suggest that the ethicist should have ethics expertise and decision-making authority or a consultative role. CEC frequently occur in acute care hospitals [5], with some programs focused on providing services in specific contexts such as intensive care units [6], pediatric areas, or end-of-life circumstances [7]. Since 2000, the rate of CEC has increased by 94% across U.S. hospitals with the median number of consults doubling in some areas. However, during this timeframe, published accounts of evaluations of CEC have decreased from 28 to 19.1% [5]. This decline is despite the fact that over the past 30 years, several journals have published special issues related to discussions about the need for evaluation and evidence of CEC quality [8, 9]. An editorial in 2017 laments the poor state of empirical studies evaluating CEC, despite national governments and health care organizations emphasizing its importance, and repeated calls from within the field for rigorous evaluation research [10–12].

The extant literature focuses on identifying the scope and expertise of ethics consultants, including ethics training [13] and credentialing [14]. Furthermore, despite some limitations, descriptive information about CEC services, including consult volumes [15], roles of consult requestors, and types of ethical issues prompting consultation is available in the U.S. and in Europe [16]. However, questions about the nature and scope of the role or roles of ethics professionals persist (e.g., are they moral authorities [17]? Are they advocates [18]?) and empirical or methodological issues continue to be debated (How do we measure morality? What is a ‘good’ consultation outcome [11]?). Studies assessing the quality of CEC are numerous [19–21]; however, some acknowledge the difficulty of measuring quality when there remains a lack of clarity and consensus about the goals of CEC and the best outcome domains to measure [22, 23]. Other studies discuss limitations posed by a lack of standardization in regard to the structure of CEC, the processes and methods employed, and the unique complexities of individual cases that inform the ethics consultant’s activities [24]. For example, a lack of standardized organizational policies that define CEC, training and education of consultants, consensus as to whether the consultant engages in a pure facilitation approach, authoritarian approach or a combination of approaches, and whether the goal of CEC is mediation, facilitation or the generation of a recommendation [25–28]. Feder and Firn argue that the CEC should “take a personalized and values-based approach to facilitating decision-making that acknowledges context and a plurality of possible ‘right’ answers” [29]. Contextual features may include the setting, inter-professional dynamics and behavior, interventionist characteristics, institutional culture, and nuances in patient cases (e.g., characteristics, diagnoses, values). Others have argued that the context-sensitive and value-laden nature of CEC is particularly challenging for developing effectiveness studies. Values-plurality and normativity of outcomes are presented as barriers to standardization and effectiveness studies; however, it is not necessarily the case that no outcome or measure can reliably be applied. An outcome may be more or less defensible because it aligns with the patient’s enduring values, or because it minimizes or averts harms that patients and clinicians agree are undesirable (e.g., pain, reduced life expectancy).

Some medical specialties have articulated positions that appear to presume which outcomes would be relevant or desired to CEC; for example, the American Thoracic Association and other critical care societies have recommended CEC as a way to prevent inappropriate treatment [30]. Although it is useful to know what outcomes would be regarded as desirable by critical care providers, it must be acknowledged that CEC serve a variety of stakeholders,^1^ including patients and families, and their values may not be congruent with those of their clinicians. Furthermore, non-beneficial treatment and cost-effectiveness have been problematized from within the field and have been viewed as unreliable outcome measures, unable to capture the nuances and complexities of the consultation and the significance of patient values [11, 31]. There is a need to identify relevant outcomes that are meaningful for all stakeholders and to develop validated measurement tools to inform future study. A well-developed tool is the EURO-MCD questionnaire, which has been validated by Dutch professionals trained in MCD facilitation, and identifies 26 moral case deliberation-related outcomes [32–34]. Further understanding of the development of this tool and associated outcomes for evaluating CEC effectiveness internationally has the potential to support and advance this important work.

Chen and Chen argue that using quantitative methods to evaluate CEC will be challenging so long as there is a lack of standardization of CEC methods or a comparator group to demonstrate an intervention’s effect [24]. However, standardization can be achieved if there is a clear definition of the scope of the intervention and agreement about the recommended approach (e.g., focus on family meetings with values-disagreement, use of a facilitation approach). Comparisons can be made to usual care; that is, care provided without consultation support. It should be noted that there are similar challenges faced in evaluating other complex health interventions, e.g. mental health strategies [35]; therefore, it should not be assumed that these challenges are insurmountable nor should it deter scientific advancement. Identifying relevant outcomes to demonstrate CEC effectiveness and developing and validating measurement tools is required. This can be done in parallel with efforts to define the nature and scope of the ethics consultant’s role with a view toward providing a foundation for robust future research, evidence-based interventions, enhanced practice standards, and quality assurance. Ultimately, these efforts can provide a sound basis for demonstrating the value of CEC.

Previous research has explored issues related to outcomes in healthcare ethics consults. For example, using a Cochrane review, researchers evaluated the effectiveness of clinical ethics supports, including CEC, in controlled studies limited to adult patients in intensive care units [36]. Other systematic reviews have assessed clinical ethics support services in the end-of-life context, intensive care units [37], or have focused on the activities of ethics committees [38]. Another topic examined through systematic review was CEC quality assessment tools [39]. There remains a need to provide an overview of the available research on the evaluation of CEC that span the range of healthcare contexts and settings in order to inform the development of a set of core outcomes for future research.

To address this gap in the literature, we undertook a scoping review to identify and map the outcomes reported in evaluations of CEC. A secondary objective of this review was to provide a comprehensive overview of CEC structures, processes, and roles to enhance understanding and to inform standardization. A scoping review is the appropriate review methodology given the paucity of existing evidence and the need to clarify key concepts, the types of evidence available, and to map the relevant outcomes across clinical care settings [40, 41]. The research question guiding this scoping review question was the following: what types of outcomes of CEC have been reported?

## Methods

**Fig. 1:**
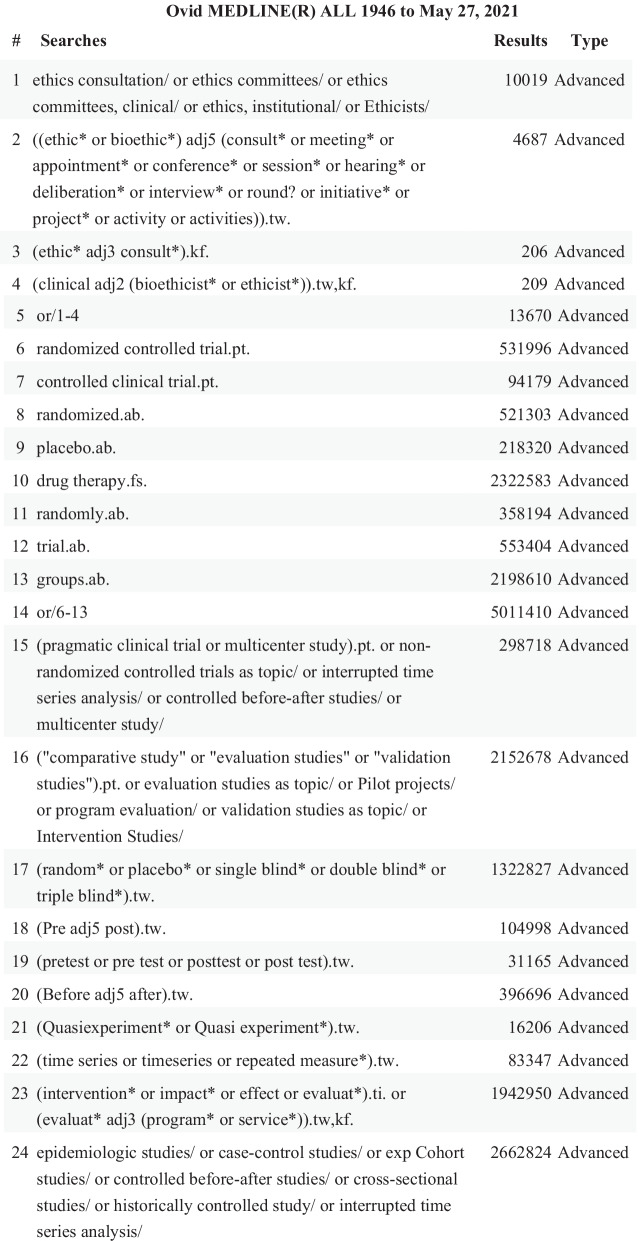

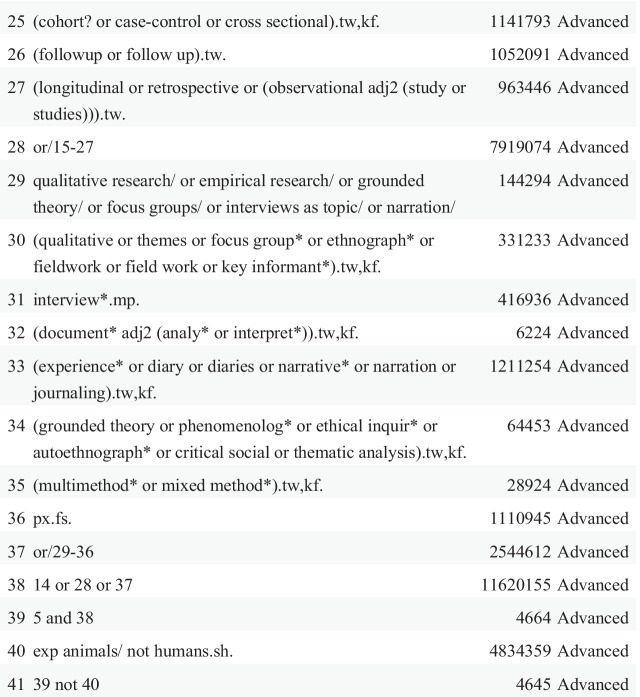
MEDLINE ALL search strategy

We sought to identify primary studies of CEC involving patients, substitute decision-makers and/or family members, clinicians, healthcare staff and leaders. We searched the following electronic databases from inception to May 2021 to identify research articles examining CEC in healthcare settings: Ovid MEDLINE ALL: Epub Ahead of Print, In-Process & Other Non-Indexed Citations, Ovid MEDLINE® Daily and Ovid MEDLINE®, OVID Embase, OVID AMED (Allied and Complementary Medicine), EBSCO CINAHL, Cochrane Central, ProQuest Philosopher’s Index, ProQuest Sociological Abstracts, Ovid Social Work Abstracts, and Ovid PsycINFO. The search strategy was developed by an academic health science librarian (EN) in collaboration with the project leads. The search strategy was translated using each database platform’s command language, controlled vocabulary, and appropriate search fields. MeSH terms, EMTREE terms, AMED thesauri terms, CINAHL headings, Thesaurus of Sociological Indexing terms, APA thesauri terms, and text words were used for the core search concept of ethics consultations and evaluation. To capture the concept of evaluation, we used a combination of search terms designed to retrieve primary studies that met our methodological criteria, including randomized and non-randomized controlled trials; retrospective and prospective observational cohort studies; and qualitative studies. When appropriate, we incorporated validated search filters; for example, the Cochrane RCT filter for Medline [42]. Finally, we applied a human filter to the Medline and Embase strategies [42]. No date, language, or jurisdiction limits were imposed. The original search was run in January 2019 and run again in May 2021 (Fig. 1).

We attempted to identify additional studies by searching the reference list of relevant studies included for full-text review, and by hand searching relevant journals identified from the database searches. In addition, we attempted to identify ongoing and/or unpublished studies by searching dissertation databases, relevant websites of professional organizations, and clinical trials registries [43]. The trial registry search did not yield any results.

Articles retrieved from the searches were exported and saved in EndNote X9 reference management software [44]. We used Covidence web-based literature review software and an in-house database for screening and data abstraction.

### Screening

The search was executed and duplicates removed according to the auto- and hand-searching methods outlined by Qi et al. [45] The research team members (JB, AH, AB, RS, KR, DB, MS) independently screened titles of studies retrieved using the search strategy and those from additional sources to identify studies that met the inclusion criteria (see Table 1). If the eligibility of a study could not be determined by the study title, the reviewers screened the abstract for relevance. The full text of studies deemed potentially eligible were retrieved and independently assessed for relevance according to inclusion criteria. Any disagreement between reviewers over the eligibility of particular studies was resolved through consultation with a third reviewer. The reviewers requested missing data from the study authors when there was insufficient information to conduct the review. Studies with insufficient information regarding intervention content (e.g., unable to determine whether the intervention was an ethics consult) were excluded from the review.

**Table 1:** Study inclusion criteria

Participants	Studies of patients, substitute decision-makers and/or family members, clinicians, healthcare staff and leaders who were involved in clinical ethics consultation(s)
Intervention/Exposure	Studies of clinical ethics consultations in a healthcare setting. For the purposes of this review, clinical ethics consultations are defined by the following consensus statement: “a service provided by an individual consultant, team, or committee to address the ethical issues involved in a specific clinical case” [4]
Comparator/Control	Studies of clinical ethics consultations including a comparison of standard care or active control were eligible for inclusion. Studies of clinical ethics consultations without a comparison group were also eligible
Study designs	Studies of clinical ethics consultations, including randomized and non-randomized study designs, observational cohort studies, and qualitative studies to identify the outcomes reported in primary studies evaluating clinical ethics consultations. Opinion articles, case series and theoretical papers were not eligible for inclusion
Context	Primary studies of clinical ethics consultations in a healthcare setting
Outcomes	We aimed to identify the outcomes reported in evaluations of clinical ethics consultations. Based on our clinical experience and preliminary searches, we anticipated that outcomes would include assessments across the following domains: 1. Psychological factors: e.g., moral distress 2. Process factors: e.g., facilitating consensus 3. Healthcare utilization: e.g., number of days admitted to hospital 4. Clinical factors: e.g., documentation of goals of care in the electronic medical record 5. Quality: e.g., satisfaction (service quality)

**Fig. 2:**
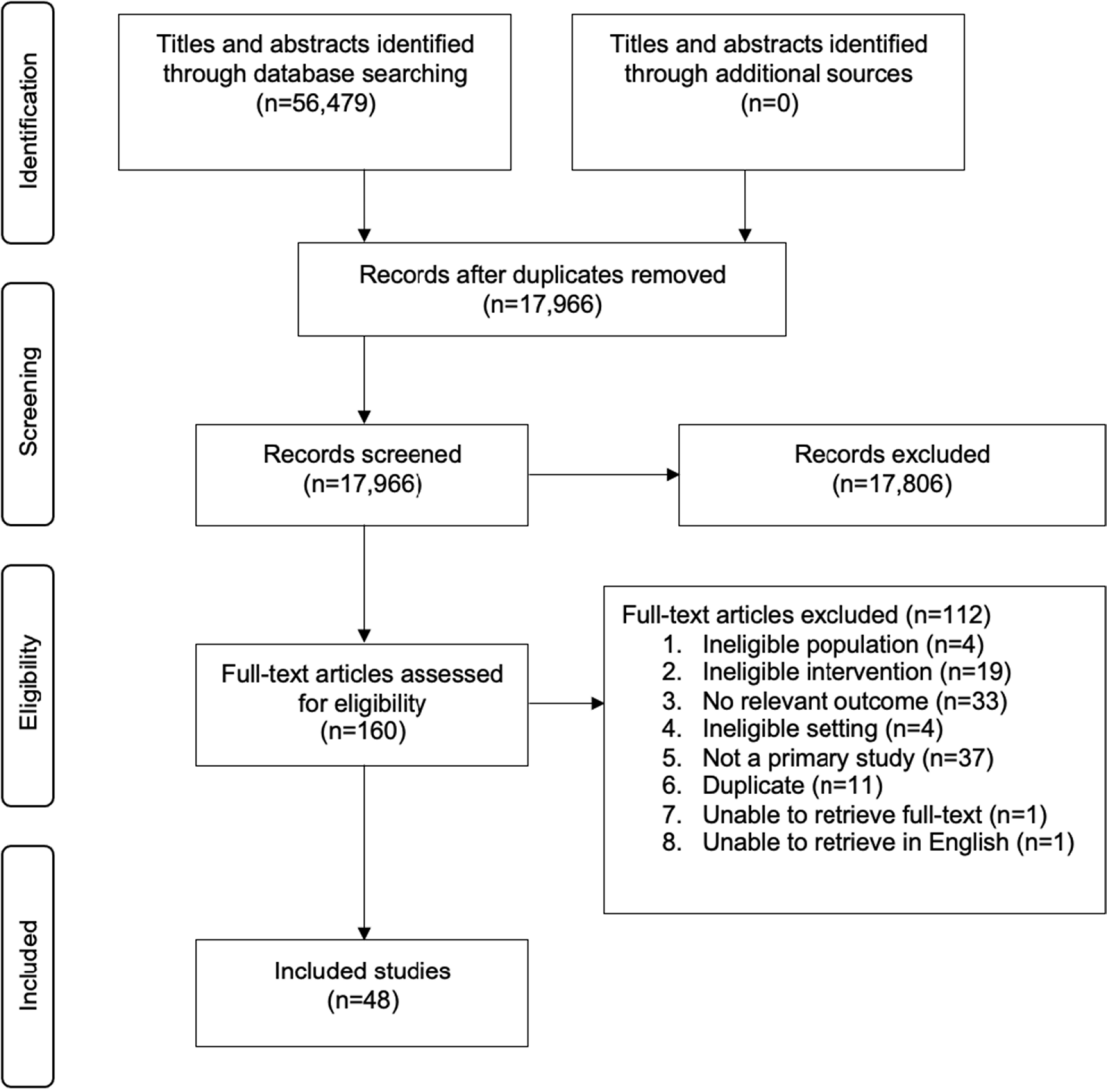
Study flow diagram

In total, our searches returned 56,479 articles. After removing duplicates and studies that did not meet the inclusion criteria, 48 articles comprised this scoping study (See Fig. 2).

### Data extraction

Data extracted from the included studies used a piloted standardized form. Extracted information included: study design; study setting; population and participants; discipline of requestor; intervention components (e.g., setting, purpose of consult, individual consultant vs. team), and outcome assessments. The first author (JB) and two other team members (ET, MS) extracted data independently and resolved discrepancies through discussion. A risk of bias assessment was not conducted as this is not applicable for scoping reviews [46].

### Data synthesis

Information was collated, summarized and reported in accordance with PRISMA-ScR reporting standards [46].

An initial set of outcomes were identified across five conceptual domains a priori based on our clinical ethics experiences and preliminary literature searches. These domains included: psychological factors, process factors, healthcare utilization, clinical documentation, and quality outcomes. The domains were then refined or revised through an interpretative process of identifying and synthesizing the outcomes identified in the scoping review literature. For example, the domain “psychological factors”, which included predominantly moral distress, was revised early in the review process to “personal factors” to capture the broad array of individual-related outcomes that studies were reporting on. Additionally, healthcare utilization was re-interpreted as the more encompassing “resource outcomes” domain to include cost considerations. The final set of outcomes that studies reported on included: personal factors, process factors, clinical factors, quality, and resource factors.

Mapping the literature occurred through an iterative process of reviewing each study and interrogating which of the domains, if any, appropriately described outcomes depicted. If a study included multiple outcomes without reference to primary, secondary, etc., each outcome presented was categorized and interrogated separately in order of appearance in the study. Categorization occurred in accordance with the primary description or emphasis, and study context. For example, a quality improvement study that discussed the perceived usefulness of the CEC in clarifying ethical issues could be categorized as representing a quality (usefulness) or a process factor (clarifying ethical issues). Since the study was a quality improvement design and attention to usefulness was the primary focus, we categorized this outcome as quality-related. In addition, some studies reported outcomes within a particular domain, but were re-categorized according to our pre-specified criteria in an effort to systematize outcome domains in the field. For example, White (1997) reported increased knowledge as related to satisfaction [47]. This was re-categorized for the purposes of our review as a personal factor since increased knowledge relates more broadly to a change in stakeholder perspective or experience. When conflicts in categorization arose, the domain classification was discussed and interrogated until a consensus was reached on its assignment. Judicious notes and a decision bank of challenging outcome domain assignments were kept in the study database for the review team to access for help in categorizing domain assignments.

## Results

### Study characteristics

Forty-eight studies were included in the review, with a majority of studies conducted in the United States (n = 27). Other countries represented were Norway (n = 4), the Netherlands (n = 5), Sweden (n = 4), Germany (n = 2), as well as n = 1 each from Canada, Taiwan, Japan, Chile, and Australia. One study was conducted across Norway, Sweden, and the Netherlands. More than half of the studies were conducted within a hospital setting (n = 27). Three studies identified the setting as a pediatric hospital. Ten studies took place in adult intensive care units, and one study took place in a neonatal intensive care unit. Additional study sites included aged care, community health and care, psychiatric outpatient clinics, mental health care institutions, in-home care settings, mobile health clinics, and transgender care. Two studies occurred across multiple settings, and one did not identify a clinical setting (Table 2).

**Table 2:** Study characteristics

Location	Setting	Study design	Study period	Population	Sample size (N =)	Reference #
United States	1 medical/surgical ICU at a large, tertiary, not-for-profit medical center	Prospective randomized exploratory trial	October 2007 to February 2010 (28 months)	English-speaking adult patients received treatment in the medical/surgical ICU for at least 5 days	384 (Intervention N = 174, Control N = 210)	[48]
Norway	9 different hospitals from six different locations in Norway	Qualitative Study	2006–2009 (36 months)	Doctors who had referred EOL decision to a CEC, or has been a member of a medical team who requested CEC assistance	15	[49]
Sweden	1 publicly funded children's hospital in Sweden	Qualitative Study	Not indicated	Healthcare professionals (physicians, nurses, nurse assistants, psychologist, and play therapists) working at hospital	6 ECR sessions observed, 35 healthcare professionals in the six ECR sessions; 10 healthcare professionals were individually interviewed outside of the ECR sessions	[50]
United States	large tertiary care facility that is both a community hospital and a major academic medical center	Quality Improvement	July 1–December 31 in 2011 and 2013 (10 months total)	Nurses who requested CEC	15	[51]
Chile	ICU at a private clinic in Santiago, Chile	Quality Improvement	Not indicated	Intensivist Physicians	26	[52]
United States	562 bed academic hospital	Quality Improvement	2011–2012 (12 months)	Staff, students, patients, families	184	[53]
Sweden	integrated heart-failure and specialist palliative in-home programme at Palliative Advanced Homecare and Heart Failure Care	Qualitative, descriptive study	Only indicates year May 2013	PREFER team (specialized care team) consisting of physicians (1 cardiologist, one GP specializing in palliative medicine), RNs (2 district nurses one heart failure palliative care nurse), OT (1), and physiotherapist (1)	7	[54]
United States	Adult ICUs of 7 US hospitals selected to represent a broad spectrum of characteristics, including community, religious, managed care, and academic institutions with diverse patient populations. All have busy ICUs and active CECs	Mixed methods	Not indicated	Healthcare providers and patients/surrogates/family members who were identified as potentially having a values conflict	363 (N = 108 family members, N = 255 HCPs)	[55]
United States	600-bed academic hospital in a large metropolitan city in the MidWest	Qualitative Study	Not Indicated	Healthcare professionals representing multiple areas of the hospital and consisted of nurses, physicians, and social workers	16	[56]
United States	Cleveland Clinic Foundation	Quality Improvement	August 1985–April 1992 (~ 80 months)	Family members and staff involved in the consultation	26 (N = 6 family members, N = 40 staff members)	[57]
Netherlands	Neonatal intensive care unit of a pediatric teaching hospital in the Netherlands	Quality Improvement	Not indicated	Multidisciplinary healthcare professionals (physician, nurse, nurse practitioners, social worker, pastor) involved in the MEDM (medical ethical decision-making)	105	[58]
Sweden/Norway/Netherlands	Community care, somatic hospital care, psychiatry, care for mentally disabled, Dutch health inspectorate, and hospital policy departments	Descriptive Longitudinal field survey and psychometric testing	Not indicated	Nurses (RNs, Support Workers, Psychosocial Workers), Nurse Assistants, Doctors/Specialists/Psychiatrists, Therapists (Physiotherapists, Psychologist, Spiritual Caregivers, and Social Workers), Managers (Department Heads and Policy Makers), and Others (Volunteers, Clients, Researchers, Trustees, Secretary's and Interns)	443 HCPs participating in 4 MCDs across 30 institutions (Sweden = 6, Netherlands = 10, Norway = 14) (T1) 247 HCP's participating in 8 MCDs across 25 institutions (Sweden = 6, Netherlands = 5, Norway = 14) (T2)	[59]
United States	ICU for terminally ill/critically ill patients at Hospital in the US	Prospective, controlled group design study	June 1992–October 1994 (28 months)	ICU patients treated with > 96 continuous mechanical ventilation	99	[60]
United States	Nonprofit tertiary hospital in Los Angeles	Retrospective analysis; quality	July 1–December 31 in 2011 and 2013 (10 months total)	Members of clinical team who requested consultation (i.e. physician, social worker, and nurse)	91 questionnaires completed by 58 individuals	[61]
Norway	Norwegian Hospitals (Number Unspecified)	Qualitative study	Period not indicated; study began in May 2004	Clinicians who had brought cases to the ethics committees for case consultation	8	[62]
Japan	Japan; as the first clinical ethics service in Japan, informed general public of service thru mass media and team's medical institution affiliations in Japan	Quality Improvement	October 2006–December 2007 (14 months)	Medical practitioners	25 consultations; 22 of which were requests from medical practitioners (N = 18 responses received)	[63]
United States	6 hospitals	Randomized Control Trial	2000–2002 (24 months)	Patients in adult intensive care unit	499 (of 551 study patients); intervention (n = 252) vs control (n = 247)	[64]
Taiwan	3 surgical intensive care units in Hospital	Randomized control trial	Not indicated	Patients in surgical intensive care units with "medical uncertainty/conflict regarding value-laden issues"	62 patients (33 randomly assigned to HCEC group; 29 randomly assigned to UC group)	[65]
Australia	Centre for Children's Health Ethics and Law (CCHEL); situated within a 359-bed, tertiary-quaternary pediatric hospital	Quality Improvement	Referrals from February 2015-January 2017. Design proceeded in three phases over a study period of 24 months	Healthcare staff (consultant medical officers, nurses, allied healthcare professionals, advanced trainee medical officers, social workers)	35 responses from 11 cases	[66]
Netherlands	Dutch organization for aged care in the south of Netherlands consists of 20 care centres	Mixed Method Evaluation study	Period not indicated; study occurred in 2009	Nursing caregivers of different educational levels, motivational therapists, dietitians, team leaders	61 moral case deliberation (MCD) sessions organized in 16 care centres; 493 questionnaires returned (team leaders N = 43, professional caregivers N = 450); 5 in-depth interviews and 3 focus groups	[67]
United States	712-bed community teaching hospital with 700 member private medical staff	Prospective Study	January 1, 1988–December 31, 1989 (12 months)	Requesting physicians and patients referred for consultation	104 requests; 83 physicians participated (80%); 104 patients	[68]
United States	University teaching hospital	Prospective study	Period of evaluation July 1, 1986 to June 2, 1987 (11 months)	Requesting physicians and patients referred for consultation	51 requests; 45 physicians	[69]
Germany	Large German general hospital with over 1700 beds and 26 specialized clinical departments	Observational study	January 2006-June 2015 (~ 103 months)	Adult inpatients in 3 inpatient settings: ICU, low care units, psychiatric care	259 CECs (ICU 43.6%, low care units 33.6%, psychiatric care units 22.8%)	[70]
Canada	No information about clinical setting; all participants were from Toronto, Canada	Qualitative	Not indicated	Family caregivers involved in making care and treatment decisions for a hospitalized family member at the end of life	20 (12 women, 8 men; 2 from the same family); only 5 participants used the services of the clinical ethicist during their decision-making process	[71]
Norway	Community Health and Care Settings	Quality Improvement	January–March 2015 (1st survey) and April- September 2015 (2nd survey(7 months)	Municipal contact persons for the ethics project (ethics activities implemented in the health and care sector in more than 200 municipalities), and ethics facilitators	137 (municipal contact persons); 217(ethics facilitators)	[72]
Norway	19 Norwegian Hospitals	Prospective questionnaire (Mixed Method)	September 2016–2017 (12 months)	Clinicians, patients, next of kin, managers, patient ombudsman, CEC members	61	[73]
United States	476-bed teaching hospital and tertiary referral center of New York Medical College	Quality Assurance (Quality Improvement)	January 1990-December 1992 (24 months)	Physicians, nurses, patients, family members who were associated with 20 sequential cases referred to CEC	61 (24 physicians; 20 nurses; 17 patients or family members) regarding 20 consultations in a two-year period	[74]
United States	Suburban community hospital	Economic Analysis	July-December 1994 (5 months)	Economic analysis of 29 patient cases/consultations	29	[75]
Netherlands	Academic psychiatric hospital	Quality Improvement	4 year project (48 months)	Participants of MCD sessions (e.g., nurse, psychologist/therapist, physician, manager/chief, other occupation)	69	[76]
United States	Department of Family Medicine at 625-bed tertiary care teaching hospital	Retrospective design (medical chart review) and physician evaluation	August 1, 1990–July 31, 1991 (12 months)	Requesting physicians	43 attending physicians	[77]
United States	Department of Family Medicine 625-bed tertiary care teaching hospital	Prospective study	February 1, 1994–January 31 1995 (12 months)	Patients, caregivers (parents, spouses, adult sons/daughters, siblings, other family member, friends)	56 interviews with patients or their surrogates (caregivers)	[78]
United States	University of Texas Health Science Center at San Antonio—includes a 630 bed county hospital and 700-bed Veterans Administration hospital	Prospective study and retrospective design	18 month period (January 1984- June 1985)	Physicians who requested consultations	24 (physician requestors) regarding 44 consultation	[79]
Germany	University hospital	Nonparticipating observation of consultations (prospective and retrospective) and subsequent qualitative evaluation	Not identified	Ethicists, physicians, nurses, psychologists	28 interviews and 14 observations	[80]
United States	Medical and pediatric ICUs in a university medical center	Prospective, randomized controlled intervention trial	February 1997–October 1998 (20 months)	Patients in whom value-based treatment conflicts arose during the course of treatment; healthcare providers and family members involved with the 23 intervention patients who had received ethics consultation	N = 70 (CEC group n = 35; UC = 35); Interviews, N = 55; n = 47 providers (28 medical doctors, 14 registered nurses, 3 social workers, 1 managed care representative, 1 chaplain), n = 8 family members	[81]
United States	Adult ICUs of 7 hospitals across the US	Multi-centre prospective randomized controlled intervention trial	November 2000–December 2002 (24 months)	Adult patients in whom value-related treatment conflicts arose during the course of treatment that could lead to incompatible courses of action; interviews with nurses, physicians, patients or surrogates who received ethics consultations	N = 551 (CEC group n = 278; UC = 273); Interviews, N = 383 (n = 272 nurses and physicians, n = 111 patients and surrogates—2 pts, 109 surrogates)	[82]
Sweden	Psychiatric outpatient clinics in a county in Sweden	Qualitative study (exploratory/descriptive design)—content analysis	Not identified	Healthcare personnel (working in 2 different psychiatry outpatient clinics with experience of participating in ethics rounds)	11	[83]
United States	The Cleveland Clinic Foundation	Quality Improvement	Period not identified, study began in Spring 1990	Health professionals who deal directly with patients and families and who are called upon to make decisions regarding appropriate patient care	794 (n = 213 staff physicians, n = 176 resident physicians, n = 370 nurses, n = 26 social workers, n = 9 pastoral care staff) [46% of respondents had used the Dept of Bioethics—consult service for the purposes of this scoping review]	[84]
United States	Medical-surgical ICU at a comprehensive 470-bed academic cancer center in New York City	Retrospective design (using ICU, hospital, and ethics databases)	September 2007–December 2011 (51 months)	Adult patients with cancer who were admitted to the ICU and who had an ethics consultation	53	[85]
Netherlands	Large mental health care institution	Mixed method (responsive evaluation method through dialogue with stakeholders) structured interviews and semi-structured questionnaire conducted after request received before start of the session and subsequent evaluation questionnaire after MCD Likert scale and open ended questions)	April 2008–April 2011 (36 months)	Managers and nurses	78 (Pre Session) and 255 (Post MCD)	[86]
United States	Nine critical and special care units at Saint Thomas Hospital in Nashville, TN—a 670 bed adult acute-care facility and a teaching hospital affiliated with a university medical center	Qualitative survey	Did not indicate	Clinical ethicist, nurses and physicians involved in the individualized CEC (the clinical ethicist participated in self-evaluation)	88 (n = 37 ethicists, n = 23 nurses, n = 28 physicians)	[87]
United States	350-bed university-affiliated community hospital in Portland, OR	Mixed Method	1993–1995 (24 months)	CEC requestors (requests were made by attendings, consultants, residents, nurses, ancillary staff, hospital administrators, medical staff committees)	45	[47]
United States	Single-site 900 + bed academic medical centre with a dedicated ECMO programme	Mixed Method (retrospective review of EC documentation and semi-structured interview)	August 15, 2018 to May 15, 2019 (9 months)	Clinicians caring for patients on ECMO	Interviews N = 20 Chart Review N = 68	[88]
United States	PICU in a quaternary care children's hospital; PICU has 26 beds	Pre/post design and using retrospective historical controls	Does not indicate	Physicians and nurses on staff in the pediatric intensive care unit; patients were identified for PEACE round discussions and historical controls	N = 126 patient cases (n = 60 CEC group, n = 66 historical control group); N = 42 ( n = 32 nurses and n = 10 physicians) completed the pre/post survey	[89]
United States	Single large academic healthcare system in the Midwest that includes two urban adult hospitals and one pediatric hospital	Survey and qualitative interview	Does not indicate	Healthcare professionals involved in the care of a patient who was the focus of a CEC, hc professionals who initiated a CEC, or who were present at a patient care conference attended by an ethics consultant	N = 115 professionals completed the survey; N = 48 interviews (n = 9 social workers, n = 22 nurses, n = 17 physicians), representing 13 consultations	[90]
United States	Pediatric teaching hospital	Retrospective chart review and structured interviews (5-point Likert scale and open ended narrative)	September 4, 1990-April 10, 1995 (55 months)	Pediatric ethics consultations; physicians, social workers, family members	N = 35 pediatric ethics consultations (chart review); n = 23 physicians or social workers, n = 4 family members, involved with 23 of the consultations (interview survey)	[91]
Sweden	University hospital (peritoneal dialysis and dialysis), general hospital (medical assessment unit), community hospital (internal medicine, dialysis, geriatric cardiology), and municipalities (rehab, short-term care)	Explorative qualitative interview study utilizing semi-structured interview	Not identified	First-line managers	11	[92]
Netherlands	2 transgender clinics offering gender affirming medical treatment (clinic at the Centre of Expertise for Gender Dsymorphia at the Amsterdam University Medical Centre; clinic at Curium-Leiden University Medical Center)	Mixed Method (qualitative interviews, focus groups and questionnaire that includes a 26 item survey and open ended questions)	Not identified	Specialists in child and adolescent psychiatry and psychology, endocrinology, pediatric endocrinology, surgeons and gynecologists	N = 6 MCD sessions were audiotaped & analyzed; N = 6 individual interviews with MCD participants; N = 2 focus groups (15 participants total); N = 34 (Pre-Session) N = 22 (Post-MCD) questionnaire	[93]
United States	A large, tertiary academic medical centre	Qualitative interview study	May 2020 to July 2020	Healthcare professionals (physicians, nurses, social workers, etc.) involved in an ethics consult between March 11, 2020 and May 6, 2020	14	[94]

The predominant study design was quality improvement^2^ (n = 13), followed by mixed method (n = 10), qualitative (n = 9), randomized controlled trial (n = 4), and retrospective/prospective or a combination (n = 10). Standalone methods included economic analysis and observational study. Twenty-eight studies identified a time period; among these the average length of study was 26.75 months. The majority of studies (n = 33) were published in 2016 or earlier; 15 studies were published within the past 5 years, with a greater proportion being conducted in European countries. The primary study population was healthcare professionals (n = 29). Few studies focused on patients (n = 7), even fewer on family members or substitute decision-makers (n = 1), and 11 studies combined two or more of these groups (i.e., patients, family members and/or health care professionals) (Table 2).

### Delivery context

The CEC intervention was most often identified as a clinical/ethics/ethics committee consultation or service (n = 34). Six studies used the term moral case deliberation. Other descriptions referenced ethics consultation system, ethics case reflection sessions, ethics rounds, clinical ethics support, structured multidisciplinary medical-ethical decision making, ethics intervention, and clinical ethicist involvement. Differences in terminology reflected nuances in understanding and delivery of CEC, including format, timing of the intervention (e.g., proactive or reactive), and processes (see Table 3). Despite the different descriptors, studies nevertheless fell under the general category of clinical ethics consultation or moral case deliberation given the similarity in structure, delivery, and purpose.

**Table 3:** CEC intervention delivery context

Name of intervention	Intervention Coded As	Description	CEC requestor	CEC deliverer	Training/experience of deliverer	Reference #
Proactive Ethics Intervention	Clinical Ethics Consultation	9 step process initiated prior to the identification of an ethical issue or request for ethics evaluation (differing from consultation) consisting of a series of encounters involving a bioethicist in the care of ICU patients with an LOS of 5 days +	No request is made as approach is proactive and defined as the point in care in which "no ethical issues have [been] identified and no request for an ethics evaluation has been made"	Standardly involves 2 staff members involving a clinical ethicist and a research assistant	The ethicist involved has completed Master's, PhD, and fellowship, and has (3) years of experience as a clinical ethicist in a major U.S. hospital; the activities of the ethicist were overseen by 3 individuals with "years of experience" in ethics consultation	[48]
Clinical Ethics Consultation	Clinical Ethics Consultation	A consultative, supportive, and educational function in which the main function is to secure that value issues are recognized and dealt with in a competent way	Physicians	Hospital Ethics Committee	Study denotes that ~ 87% of informants (CEC deliverers) had familiarity with CEC whereas remaining deliverers had only recently experienced CEC	[49]
Ethics Case Reflection Sessions	Clinical Ethics Consultation	Similar structure to ethics rounds in which an ECR session is organized as meetings involving the interprofessional team and the external facilitator	Interprofessional	External facilitator	Article does not discuss training/credentials of the EC < but does denote training/experience as a potential limitation	[50]
Clinical Ethics Consultation	Clinical Ethics Consultation	An “individual but tethered” approach in which the individual consultant on the case remains in consultation and communication with other CHE faculty members, but is assigned to the case individually	Nursing	Individual Ethics Consultant	Article does not discuss the training/credentials of the EC	[51]
Ethics Consultation System	Clinical Ethics Consultation	Vague description, but the individual consultant coordinates work with the ethics committee	Treating Medical Team or by relatives	Individual Ethics Consultant	Does not identify training/credentials of the EC	[52]
Ethics Consultation Service (ECS)	Clinical Ethics Consultation	Individual intervention performed by consultant, followed by a weekly, hour-long ethics case review conference to review recent consultations. For complex consultations, discussion with the Healthcare Ethics Committee occurs	Does not explicitly specify but indirectly alludes to staff and family as options	Individual Ethics Consultant	ECS staffed by 2 clinical ethicists who combined have 40 + years of clinical practice experience, each having completed advanced degrees and mentorship in ethics	[53]
Clinical Ethics Support	Moral Case Deliberation	Interprofessional reflection on ethical dilemmas encountered in clinical work conducted through scheduled interprofessional meetings held every third month. A 60 min author-led session began with open-ended requests to the team members who thereafter collectively decided on the situation of discussion. Each participant reflects on the situation and the ethical issues involved. A value conflict is identified, and suggestions for response/resolution are discussed	No request is made due to nature of intervention	CES Leader	Leader belongs to department of nursing; facilitator “may be healthcare professional or even a philosopher-ethicist” with the role of promoting ethics dialogue	[54]
Proactive Ethics Consultation	Clinical Ethics Consultation	Consults offered in response to latent or manifest conflicts rather than specific consultation requests. Adhered to a general process model including review of the medical record, discussion with healthcare team and family, assessment of issue, timely meetings as appropriate, and recommendation for next step	No request is made due to nature of intervention	Individual Ethics Consultant	Article does not discuss the training/credentials of the EC	[55]
Hospital Ethics Committee Consultation Service	Clinical Ethics Consultation	Members of the service evaluate the clinical situation through interviews with healthcare professionals,	The sample was not selected based on having requested a CEC. Healthcare professionals who had been involved in a particular ethics consultation case were recruited as part of a larger online study. A subset of these participants participated in the qualitative study	Hospital Ethics Committee	Article does not discuss the training/credentials of the EC	[56]
Structured Multidisciplinary MEDM (medical ethical decision-making)	Clinical Ethics Consultation	All participants but the chair (primarily responsible for the process) are directly involved in caretakers’ (differs from consultations in that they are consulted in complicated/exceptional situation requiring external expertise).Sessions are scheduled when the provider/parent have doubts about moral justification of a child’s treatment. Meetings are chaired by an impartial ethicist. Follows 5 steps (same steps for deliberation then reporting): (1) e*xploration* of elements to be considered, presented by representative of every professional group involved; (2) *agreement* on ethical dilemma/investigate possible solns; (3) *analysis* of solns; (4) *decision-making*; (5) *planning actions* (e.g., who will inform parents. scheduling next meeting, guaranteeing child's comfort	Not specified but indicates that sessions scheduled when provider/parent expresses doubt about moral justification	Chair of MEDM	Does not identify credentials other than chair being an ethicist	[58]
Hospital Ethics Committee Consultation Service	Clinical Ethics Consultation	Specific steps include data gathering and issue identification by a staff bioethicist. Team meets as a committee first and then meets with individual healthcare providers. Consultant discusses with patient and the family or surrogates, and the HCPs. Deliberation than occurs by the committee	Does not identify requestor	Hospital Ethics Committee	Does not identify training/credentials of the ECs	[57]
Moral Case Deliberation (MCD)	Moral Case Deliberation	A group dialogue in which professionals (sometimes with patients and families) jointly investigate a moral question emerging from a situation experienced in daily practice led by a trained facilitator	Participants specifically recruited to participate in MCD for purpose of study	Trained Facilitator	Does not identify training/credentials of the ECs	[59]
Proactive Ethics Consultation	Clinical Ethics Consultation	A consultation provided to the clinical team to increase the team’s attention to decision-making and communication process issues; consultation had both structured and unstructured dimensions	Not applicable due to nature of intervention	Two clinicians	Specifically trained in clinical ethics	[60]
Clinical Ethics Consultation Service (CECS)	Clinical Ethics Consultation	Individual but tethered style where staff individually engages with key participants associated with a given ethics consultation, but that individual remains in communication and consultation with other CHE faculty members throughout the process	Anyone in patient’s care can request including physicians, nurses, social workers, other staff, patients, and families t	Individual Consultant from Faculty of Centre for Healthcare Ethics	Does not identify training/credentials of the ECs	[61]
Clinical Ethics Committee	Clinical Ethics Consultation	Case is brought to the committee and is subsequently deliberated on by members	Evaluated clinicians who brought cases forward to the committee for consultation	Can be delivered either by committee alone, or CEC in conjunction with professionals involved in the case/on the ward	Article does not identify training/experience of ECs, but does identify that members are interdisciplinary and identifies *exclusions* from the process including patient-representatives, lawyers, and ethicists	[62]
Clinical Ethics Consultation Service	Clinical Ethics Consultation	Cases are sent by email/fax to front office of the program. The front office removes identifying info and passes onto the consultation teams who collectively formulate advice for the case and reply within a week	Medical team, ethics committee, physicians, families, nurses	Three person consultation team	17 volunteer educators/researchers including scholars of biomedical ethics, philosophy/ethics, legal, nurses, and doctors	[63]
Ethics Consultation	Clinical Ethics Consultation	General steps included (1) medical review, (2) ethical diagnosis, (3) recommendations of next steps, (4) documentation of consultation in patient's medical record, (5) follow-up by ethics consultant. No standardized protocol across hospital sites	Not applicable as participants randomized to either received consultation (intervention) or not (control)	Individual Ethics Consultant	“Trained, experienced medical ethics consultation service” comprised of individuals skilled in facilitating communication, and are knowledgeable in ethics and the law, and are officially backed by an institutional ethics committee	[64]
Health Care Ethics Consultation (HCEC)	Clinical Ethics Consultation	Case consultation following the bioethics consultation taskforce: (1) gather relevant data; (2) clarify relevant concepts; (3) clarify related normative issues; (4) help to identify a range of morally acceptable options; (5) facilitate consensus	Physicians and Nurses	Individual Ethics Consultant	Listed as having doctoral degrees, > decade of training in clinical medicine, > 20 h of clinical ethics educational courses and years	[65]
Clinical Ethics Consultation Service (CECS)	Clinical Ethics Consultation	Facilitative model to assist clinical team's decision making. Referrals accepted by consultant/fellow to decide on appropriate response Level 1: attendance by fellow/consultant at multidisciplinary team mtg to identify/clarify ethical concerns Level 2 (more complex cases): multidisciplinary clinical team and CECS response team (> 3 members) meet, chaired by fellow/clinical lead, committed to convening within 48 h of referral	Does not specify but model is to assist clinical team in decision making and responding to the clinical team specifically	Three Person team consisting of clinical lead, pediatric fellow, and administrative officer	CCHEL consisted of medical specialist clinical lead, pediatric fellow, and administrative officer. Clinical lead requires postgraduate training in ethics and fulfilment of core competencies listed in study consisting of *knowledge* core competencies, and *skill* core competencies (see Table 2 of article). Fellow advanced training in pediatrics and selection criteria requires postgraduate studies in ethics and core competencies are developed under supervision of clinical lead. Recruited by expression of interest and members are required to have some, but not all core competencies listed in Table 2	[66]
Moral Case Deliberation (MCD)	Moral Case Deliberation	Structured deliberations using either a dilemma conversation method or a Socratic dialogue conversation method facilitated by a trained facilitator	Not applicable	Trained MCD Facilitator	Individuals involved in dissemination of MCD process consisted of location manager, spiritual caregivers, and social workers and help was sought from an academic expertise group in clinical ethics support; MCD facilitators (17 individuals) trained specifically to step into this role—specific training/roles/experience of facilitators not discussed outside of denoting specific training from aforementioned individuals to step into this role	[67]
Formal Ethics Consultation Service	Clinical Ethics Consultation	Responding to the physician requests, seeing the patient, speaking with the involved parties, making recommendations, writing a formal report in the medical record	Physician (internal, family, geriatrics, surgery, psychiatry, pediatrics, obstetrics, emergency)	Internist	102 consultations performed by a board- certified internist who *completed a fellowship in clinical ethics;* there are no other formal mentions of training/experience, but study does mention "physician-ethicists" suggesting some background in ethics	[68]
Clinical Ethics Consultation (CEC)	Clinical Ethics Consultation	4 step consultation procedure: (1) elaboration of ethical question; (2) gathering of data/info from CEC participants, (3) identification/discussion of ethical arguments, (4) recommendation on course of action Uses a semi-structured protocol and documented in patient record	Discipline of requestor not indicated	Trained facilitator	Training in clinical ethics	[70]
Formal Ethics Consultation Service	Clinical Ethics Consultation	Consultant interviewed/examined patient, reviewed medical record and interviewed requesting physician/other HCPs in circle of care/family members, consultant and attending physician ethicist wrote assessment and recommendations in medical record	Physician (internal, surgery, neurology, neonatology, pediatrics, geriatrics, dermatology)	Consulting team	Members of the consulting team were health care professionals that had "expertise in clinical ethics", were completing an *ethics fellowship,* and resource persons had expertise in law and moral philosophy	[69]
Clinical Ethicist Involvement	Clinical Ethics Consultation	Does not address delivery	Not clearly indicated	Clinical Ethicist	Experienced in identification, analysis, and resolution of ethical issues encountered by patients at the bedside, often being due to conflict of values	[71]
Clinical Ethics Support Structures	Clinical Ethics Consultation	Venues for ethical discussion; most commonly municipalities had established "ethics reflection groups" in nursing homes, home-based care, and sheltered housing. In an ERG, professionals typically bring their own actual cases to be discussed among the colleagues. Sessions usually last 30–90 min	Not indicated	Ethics Consult Facilitator	Does not identify training/experience of ECs	[72]
Clinical Ethics Committee Consultation	Clinical Ethics Consultation	Does not discuss in depth but identifies clinical ethics consultation provided by a committee	Clinicians, patients, next of kin, managers, patient ombudsman	Clinical Ethics Committee	Does not identify training/experience of ECs	[73]
Ethics Case Consultation	Clinical Ethics Consultation	A team of three members that rotates monthly. New consultants attend the medical ethics course offered to medical students at New York Medical College and then function as an observer on the consultation team prior to becoming a primary team member. A review of the chart is completed, then interviews with the patient if capacitated, appropriate family members and staff. A written assessment of the case that includes documentation of the facts, an analysis of ethical issues, conclusions and recommendations is prepared	Of the 20 consults (most cases involved the withholding or withdrawing of therapy), 15 were requested by physicians, 1 by a patient's family, 2 by nurses, and 2 by a hospital administrator. In general, a CEC may be requested by the patient's attending physician, house staff, nursing staff, social workers connected with the case, the patient, family members or proxies	Individual Ethics Consultant	Team always consists of one *physician*, while other members may be nurses, social workers, nutrition specialists, clergy, or train members of the general community. No specific training/experience discussed	[74]
Clinical Ethics Consultation Service	Clinical Ethics Consultation	This study does not specifically address the delivery	Patient’s primary physician	Ethicist/Ethics Consultant	Denotes some experience/familiarity with the field. No training/experience specifically mentioned	[75]
Clinical Moral Case Deliberation	Moral Case Deliberation	Meeting with an average of 10 HCPs who systematically reflect on moral issues that emerge in a clinical case they have experienced. Reflection takes 45 min—"one day" and is facilitated by ethicist and structured by a "means of conversation method" which is selected to suit the specific goal(s) of the deliberation. The role of the ethicist is to facilitate rather than give substantial advise nor morally justify/legitimize a decision	Not applicable due to nature of the intervention	Ethicist	Facilitated by a senior ethicist	[76]
Ethics Consultation Service	Clinical Ethics Consultation	Consultant discusses case with requestor; reviews chart and talks individually with members of care team, patient, family; writes consultation report in patient's chart including ethical analysis/discussion/recommendations	Physicians (pediatrics, medicine, family medicine, surgery, gynecology), nurse, medical student, chaplain	Ethicist	Provided by a family-physician-ethicist who spends his time teaching and practicing family medicine, teaching in clinical ethics, and providing consultation	[77]
Ethics Consultation Service	Clinical Ethics Consultation	Ethics consultation	Any member of the healthcare team or from patients and families	Three Clinical Ethicists	Does not discuss training/experience of ECs	[78]
Ethics Consultation Service	Clinical Ethics Consultation	The bioethics committee provides a 2-step consultation service. 1st the committee chair reviews the pt's chart within 24 h & interviews the pt, family, physicians, other caregivers to identify the ethical issues. The chairman then meets with the requesting physician to suggest ways for resolving the issue, and writes recommendations in the chart. 2nd step the bioethics committee reviews the consultation at its bi-monthly meeting to develop a consensus about managing similar cases in the future	Physician	Ethics Committee	Committee includes 8 physicians and eight nonphysicians chosen given their *interest,* and their *ability* to promote patient interests. One of the authors (HSP) chairs the committee and is an internist with fellowship training in bioethics	[79]
Clinical Ethics Committee Consultation	Clinical Ethics Consultation	In case of moral conflict, involved individuals request support from CEC. At least 2 CEC members decide whether a request is suitable to be discussed by consultation. If appropriate, consultation is scheduled. All individuals are invited to state perspective and CEC members moderate discussion	Anyone involved in the conflict include staff, patients, and/or relatives	Unclear whether provided by team or individual	All members trained according to curricula standards of the Academy of Ethics in Medicine including theoretical/practical training pertaining to teaching ethics, organisation, and the process of counselling; advanced courses are available; usually one moderator is an Ethicist	[80]
Ethics Consultation	Clinical Ethics Consultation	Does not specify, but provides ethics consultation. Unclear whether such is provided by a team or the individual consultant	ICU nurses asked to identify patients in whom value-based treatment conflicts arose. Patients were then randomly assigned to CEC or control group	Ethics Consultation Service Members	If consultation offered/provided, was done by one of four members of ECS who "qualified at the advanced level of skills and knowledge consistent with that later recommended by the American Society for Bioethics and Humanities Core Competencies for Healthcare Ethics Consultation"	[81]
Ethics Consultation	Clinical Ethics Consultation	Each site followed a general process model of CEC which involved (1) consultation, (2) assessment of request, (3) ethical diagnosis, (4) recommendations, including further meetings, (5) documentation, (6) follow-up, (7) evaluation, (8) record keeping	ICU nurses asked to identify patients in whom value-based treatment conflicts arose. Patients were then randomly assigned to CEC or control group	Individual or Groups whose training and experience correspond to the advance levels of skills and knowledge recommended by ASBH Core Competencies	Consultations provided by individuals or groups whose "training and experience correspond to the advanced level of skills and knowledge recommended by the American Society for Bioethics and Humanities Core Competencies for Healthcare Ethics Consultations"	[82]
Ethics Rounds	Clinical Ethics Consultation	Monthly ethics rounds (1 h each) for 6 months led by a moderator to help participants identify ethical problem discussed and clarify perspectives/arguments	Not applicable due to nature of intervention	Moderator	Leader of ethics rounds is a philosopher/ethicist	[83]
Case Consultation	Clinical Ethics Consultation	Medical model: written memorandum containing ethical analysis and opinions about the cases were sent directly to primary physicians. Consultations handled primarily by the department of bioethics. The Ethics Committee handled primarily but not exclusively policy	Primary physicians or their designates	Department of Bioethics	3 full-time bioethicists and two yearly bioethics fellows; no explicit mention of training/experience	[84]
Ethics Consultation	Clinical Ethics Consultation	Upon initiation of CEC the consultant identifies the relevant clinical staff, patients, and surrogates and meets with them to ascertain the issues prompting the EC and try to reach agreement on the plan of care. EC are documented in the medical record. CEC are conducted by 1 or 2 members of a sub-group of the institution's ethics committee	Any healthcare provider, patient, or family member	Individual ethics consultant	Consultants have training in medical ethics; most consultants have clinical experience of more than 10 years in ethics	[85]
Moral Case Deliberation	Moral Case Deliberation	A group of HCPs gather to deliberate on a moral case in their work in which the starting point is an actual experience. The group reflects on this, and the discussion is facilitated by a "specifically trained conversation facilitator" MCDs are facilitated only once for a number of sessions or in ongoing groups, coordinated and facilitated by the hospitals MCD steering group consisting of five professionals	Requests typically come from managers	MCD Conversation Facilitator	Denotes that steering group members come from "various segments of the organization" and in-company training for future MCD conversation facilitators is provided	[86]
Clinical Ethics Consultation	Clinical Ethics Consultation	Nurses identify cases, ethicist reviews charts to determine additional questions that might warrant a complete EC, ethicist contacts the physician on record and explores options in helping with the issues identified. Where possible, ethicist initiates conversations with patients/families to ensure understanding of information conveyed to them by physicians/nurses/professionals. Ethicists attempts to ensure thoughts, feelings, etc. of patient/family are conveyed to the healthcare providers. Where possible, ethicist organizes patient care conferences that include the patient, family, surrogate, health care providers to identify issues needing decisions and to focus attention on reasonable options and likely outcomes. Chart notes are written by the ethicist to record all patient/family contacts once a consult is received	Nurses	Two Clinical Ethicists	One clinical ethicist has a PhD in philosophy and 20 + years of clinical ethics experience; the other ethicist is a doctoral candidate in philosophy with 4 years of clinical practice experience	[87]
Ethics Consultation	Clinical Ethics Consultation	CEC service consisted of 2 physicians and a multidisciplinary team of consultants. 3 levels of consultation provided: (1) involves answering a relatively simple question by a single member of the consultation service; (2) involves facilitating a decision-making process with the healthcare team, patient and/or family, and is performed by the EC on call; (3) used for ethical dilemmas and involves a full multidisciplinary ethics consultation service review, facilitated by the on call EC. Each consultation is documented on an intake form	Members of healthcare team (physicians, residents, nurses, ancillary services) patients, or family	Various levels provided by individual consultant or combination of 2 physicians and a multidisciplinary team of consultants	2 physicians certified in medical ethics; Consultants completed 6 month educational course in ethics consultation and participate in continuing education	[47]
Early Intervention Ethics Consultation	Clinical Ethics Consultation	Routine EC instituted for patients within 72 h of cannulation on ECMO delivered by a single consultant for the duration of the case. EC is thoroughly recorded in the record and the quality of the programme is supported by an interdisciplinary medical ethics committee	Not applicable due to nature of intervention	Individual ethics consultant	Denotes that they are "certified ethics healthcare consultants (HEC-C)" with 25 + years of experience (a palliative care physician, and a professional healthcare chaplain) with additional academic training in bioethics and clinical consultation Committee members are from diverse disciplines who have served on the committee for a long period of time and/or have completed clinical ethics fellowships or bioethics masters’ programs	[88]
Proactive Clinical Ethics Consultation	Moral Case Deliberation	"PEACE" (pediatric ethics and communication excellence) rounds. Ethicist used probing questions to uncover situational risk factors/early indicators of ethical conflict. Ethicist provided just in time education and coaching on effective communication about sensitive topics; prompted team members to discuss potentially difficult ethical aspects of management, explore rationale for limiting treatment, consider consensus around survival, and make specific recommendations about code status and goals of care	Not applicable due to nature of intervention	Individual Ethics Consultant	Does not discuss the training/credentials of the EC	[89]
Ethics Consultation Service	Clinical Ethics Consultation	Ethics facilitation approach to CEC focused on supporting key stakeholders to appreciate the perspective of others, elucidating the ethical issues, and improving communication	Anyone involved in the care of patients (including patients and families)	Interdisciplinary Team	The service is a volunteer interprofessional team including physicians, nurses, social workers, lawyers, chaplains, pharmacists, and hospital administration with varying levels of training and experience	[90]
Ethics Consultation	Clinical Ethics Consultation	Consultant reviewed medical chart, examined patient if appropriate, and talked individually with members of medical team and family. After evaluation, consultant entered formal consultative report in chart, including assessment, ethical analysis, and recommendations. Follow-up meetings with HC team member and family arranged as needed. Cases were presented to hospital ethics committee. Self-described method of consultant was a dispute resolution following a stepwise approach	83% (of 23 CEC) were requested by attending physicians, two in conjunction with the parents of the patient, and one with a social worker. Two (9%) consultations were requested by parents alone, and one (4%) by a social worker alone. One consultation was requested by the pediatric house staff as part of an ethical case conference	Individual Ethics Consultant	Singular consultant described as a "middle-aged, white, male board certified internist with medical ethics training as a visiting professor and scholar at several ethics centers."	[91]
Moral Case Deliberation	Moral Case Deliberation	Sessions used the MCD dilemma method, which consists of 10 steps and lasted between 50–116 min	Board overseeing transgender clinical care in the Netherlands introduced MCD to complement the two team's regular clinical care and decision-making processes	Certified MCD Facilitators	The MCD sessions were led by trained and certified MCD facilitators employed by the Department of Medical Humanities of the Amsterdam University Medical Centers	[93]
Moral Case Deliberation	Moral Case Deliberation	Interprofessional workplace meetings led by an external facilitator who helped staff to reflect systematically on concrete ethical issues. Consisted of 5–12 participants held monthly for 8 months, lasting 60–90 min	Not applicable given intervention method	External facilitator	Does not discuss credentials of facilitator	[92]
Clinical Ethics Consultation	Clinical Ethics Consultation	Small teams of funded faculty ethics consultants; not discussed in this paper, but described in additional publication authors cite in paper	Healthcare professionals (physicians, nurses, social workers, etc.)	Ethics Consultant	only denotes that they "meet healthcare ethics consultation criteria"	[94]

Twenty-nine interventions were described as involving an ethicist(s) or ethics consultant(s), six involved an ethics committee, and 13 involved a facilitator or healthcare professional with no formal ethicist title. Of the studies that described credentials or qualifications, the majority of CEC deliverers were described as having some training, education, or certification in ethics, ethics consultation, and/or moral case deliberation (n = 9). Other experience or training included postgraduate degrees (n = 5), with some specifying doctorate (n = 3) or master’s degree (n = 1), fellowship (n = 4), clinical experience (n = 5), training in medicine (n = 3), ethics teaching experience (n = 2), non-ethics specific skills training (n = 3), and “familiarity”, “knowledge,” or “expertise” with ethics (n = 4). Some studies only identified professional background (e.g., lawyer, philosopher*).* Twenty-five studies (52%) did not discuss or were not clear with respect to the training, credentials, or experience of the ethics consultant(s)/facilitator(s).

Twenty-six studies reported that the intervention began upon request. Physicians were most frequently identified as requesting the consult (n = 15), followed by relatives and family members (n = 10), nurses (n = 8), patients (n = 8), and others (n = 6). Twenty-two studies did not identify the requestor, or a requestor was not applicable given the study design or CEC delivery. Some studies described the purpose of the interventionist as delivering a recommendation*,* whereas others described the primary role as facilitating a discussion without offering a particular solution. For example, while Molewijk et al. described the role of the ethicist as, “that of a facilitator who does not give substantial advice and does not morally justify or legitimize a specific decision” [76], Wocial et al. described one of the goals as making specific recommendations [89]. Additionally, some studies described the process as involving stakeholder engagement, whereas a few studies described a more solitary process of deliberation. For example, Orr and Moon referred to the intervention as involving a consultant who discusses the case with the requestor and individually with members of the care team, patient, family, and others involved in the conflict [77]. Smith et al. identified the process as involving a written memorandum containing an ethical analysis and opinions about the case with no external deliberation [84]. For a full description of the interventions, see Table 3.

### Outcome domain reporting

The top three outcome domains that studies reported on were quality (n = 31), process factors (n = 23), and clinical factors (n = 19)**.** The majority of studies examined multiple outcome domains. All five outcome domains were multidimensional and included a variety of subthemes (see Table 4).

**Table 4:** Outcome domain reporting

Outcome domain	Subthemes identified	Subtheme descriptors (thematic summary of measures and outcomes)	# of Studies	Reference #’s
Quality	Overall Experience	Participants' perception of the *quality* of their overall experience of the intervention as a whole, or the facilitator of the intervention including whether such was positive or negative, what the perceived benefit or purpose of the intervention/deliverer was, if they were satisfied overall, the experience of patient/next of kin involvement, and general recommendations for improvement	6	[49, 73, 76, 82, 83, 93]
	Effectiveness	Participants experience of benefit of involvement in the intervention and/or their agreement or disagreement with the ability and degree of the intervention to meet aims/measured of quality and efficacy	2	[58, 87]
	Usefulness	Evaluation of the *practical value* of the intervention including the extent to which goals are met during the intervention, whether the outcomes or results are helpful to participants, or ways in which participants appreciated the consultation or understood its purpose for their roles	16	[55, 57, 61, 63, 67–69, 73, 74, 76–78, 80, 81, 84, 91]
	Satisfaction	Participants *satisfaction* with either the overall experience or a particular constituent of the intervention for current practice, and for the future of practice including whether they themselves would seek out CEC again or recommend it to others, the ability for the intervention to meet its actual or perceived aims and purpose(s), the amount of agreement/consensus between stakeholders, or their overall impression of the intervention	11	[48, 51, 51–53, 62, 66, 73, 47, 89, 94]
	Ability to Better Practice	The ability of the intervention to improve or better practice including the betterment of practice as a whole, or improvement in patient care, handling of ethical dilemmas, employee cooperation, service quality, relationships with stakeholders, and/or work environment	1	[72]
Personal	Clarity	Did the stakeholder experience a change in the clarity of ethical issues	1	[79]
	Moral Distress	Did the recipient experience an impact of acute moral distress after the intervention	2	[66, 89]
	Confidence	Did participants (physicians) experience an increase in confidence in the final plan	1	[79]
	Learning	How much did participants perceive to have been taught or learned	2	[79, 93]
	Perceived Value/Outcomes	What was the perceived value for self and for individual practice, impact on values, perspective and/or whether stakeholders experienced any outcomes at all during the session or in their practice	3	[59, 76, 90]
	Experience	What was the general use to recipients of CEC, and whether the intervention met expectations, and in what ways did the intervention allow recipients to meet in an ethical “free-zone”	2	[54, 90]
Process	Consensus/Integration	Whether the intervention achieved consensus between or within stakeholder groups, including whether groups followed recommendations, whether options or goals of care were agreed upon at the end of consultation, whether individuals generated common care goals, and whether familial perspectives were integrated into decision making	3	[50, 65, 71]
	Adherence	The ability of the service/deliverers to adhere to service-level standards for stakeholders and whether such improved staff competency to adhere to guidelines	1	[66]
	Purpose/Impact	What was the perceived purpose or impact of the intervention on stakeholders, including the impact on their expectation of service, what the perceived outcomes were, including whether it clarified ethical issues, educated, increased confidence, facilitated decisions, whether the process was consistent with goals, respected values, resolved issues, created cooperation, developed critical attitudes, empowered, enhanced, provided understanding, boundaries, facilitated quality care, and explored policy, paradigms, and vision. Studies also examined what particular actions the intervention took to serve stakeholders	14	[49, 61, 62, 67, 78, 81, 82, 86, 88, 90–94]
	Identification	Did consultants identify issues not seen by requestors	1	[79]
	Advancing Care	Did the intervention assist in or facilitate transitioning the patient out of hospital	1	[56]
	Helpfulness	The impact of the intervention on practice including its ability to assist in identifying, analyzing and resolving issues, educating stakeholders, facilitating discussion and sharing of personal views, and whether it was perceived to have a positive impact on the case at hand	2	[55, 87]
	Support	Was the intervention perceived to facilitate support for interpersonal relationships and interactions among staff, family, and patients	1	[51]
	Clarification	Did the intervention enhance ethical reflection, increase interprofessional understanding, better ground decisions, or increase unity among stakeholders	1	[50]
Clinical	Consensus	Was there consensus regarding recommendation or agreement on goals of care	2	[70, 85]
	Patient Management and Provision of Care	Did the intervention impact the amount or kind of care patients received including the presence of orders/decisions (DNR, withholding/withdraw orders, life-sustaining treatment, limits of care, requests for spiritual care, social services, and pain management), the provision of palliative care or chaplaincy services, and whether there was agreement with the decision, a change in treatment plan or a change in patient management post-intervention	11	[55, 60, 68, 69, 77, 79, 85, 47, 90, 91, 93]
	Quality of Care	Was there a tangible improvement in the quality of patient care	1	[89]
	Coercion	Does CEC lead to lesser use of coercion	1	[72]
	Nonbeneficial Care	Did CEC impact the use or degree of use of “non beneficial care” including impact to the number of days in hospital or ICU specifically, or the number of patients using life-sustaining treatment in those who died before discharge	3	[48, 81, 82]
	Suffering	What was the amount of perceived patient suffering from provider/patient/surrogate perspective pre and post intervention	1	[48]
	Mortality	Did CEC increase/decrease patient mortality, the number of patients that died in hospital, or discharge status (dead/alive)	3	[48, 65, 82]
Resource	Length of Stay/Resource Consumption	Assessment of CEC intervention impact on length of stay or number of inpatient days in hospital or ICU specifically	4	[48, 60, 64, 65]
	Cost	Impact to overall patient-specific, departmental, or organizational cost including the total cost of stay, net cost of consultation, or impact to charges for patients	3	[48, 60, 64]
	Cost Avoidance	Expenses that were avoided or added for patients who received consultation, and the total cost avoidance given intervention	1	[75]

#### Quality

Quality was the most frequently reported outcome domain (n = 31). This domain captured the quality of the CEC, consultant, and/or overall stakeholder experience as it related to perceived usefulness, satisfaction, timeliness, accessibility, and overall benefit. Quality-related outcomes were primarily measured by survey (n = 22), followed by qualitative interviews or focus groups (n = 7) and mixed methods (n = 6).

Usefulness was the most frequent construct measured and reported (n = 16). This construct referred to the perceived usefulness of the CEC in informing practice, the perceived overall benefit of the CEC, the extent to which the CEC was beneficial in assisting patient care, the importance for physician education and medical treatment, whether a CEC would be used in the future, effectiveness in providing emotional support, mediating disputes, and clarifying ethical issues, and improving communication (see Table 5). Other prominent quality subthemes included satisfaction (n = 11), overall experience (n = 6), effectiveness (n = 2), and ability to improve practice (n = 1).

**Table 5:** Quality reporting

Quality factors assessed (name construct)	Outcome description	Outcome measure	Results	Reference #
Overall Perception of CEC Experience	Positive Experience, Negative Experience	Qualitative Interviews	12 doctors reported positive experiences of the CEC discussion; 3 doctors reported negative experiences. Positive experience was described as related to ability to scrutinize problem from an interdisciplinary perspective, easier to reach a decision after CEC discussion, CEC discussion made decision making process more well founded, got more moral and legal backing for their final decision, and viewed CEC as an important contribution to quality of their decision and increased acceptance of their decisions by disagreeing minority within the medical team. Negative experiences related to lack of systematic structure in discussion, lack of ability to scrutinize the ethical problem or add new perspectives; had to wait too long before CEC could discuss the case	[49]
Satisfaction	Level of agreement between nurses’ retrospective responses to the questionnaire and reason why CECS was involved	Mixed methods (Retrospective CES documentation review and questionnaire, including Likert-scale and open-ended/free text questions)	"The close correlation from nurses' retrospective responses seems to indicate, at the very least, that a fairly high percentage of those requesting CEC were satisfied with the service provided…"	[51]
Satisfaction	Satisfaction regarding 6 aspects: Usefulness for decision-making, contribution to better perceive ethical aspects, support to doctors, benefit for patients, support to relatives, opportunity to request ethical consultations	Survey (Likert-scale survey “total disagreement” to “total agreement”)	Consultancy was considered useful for making complex decisions (6.3/7), support for doctors (6.5), improves ability to perceive ethical aspects (6.0), benefit to patient (6.3), support for family (6.7), request occurred in timely manner (5.2)	[52]
Satisfaction	Satisfaction regarding service quality (e.g., clarification of goals of care, improved understanding, timeliness, accessibility, clarification of questions)	Mixed methods (5-point Likert scale "strongly disagree" to "strongly agree"; free-text comments/feedback)	86–92% of respondents responded positively (agreement or strong agreement) to outcome-related questions; qualitative themes that came from free input: (1) timeliness and accessibility of the CEC; (2) clarification of the patient's goals for care; (3) helpfulness of the CEC to staff and family members; (4) appreciation for the professionalism and compassion of the ethics consultants	[53]
Usefulness and Satisfaction	Value in terms of helpful, informative, supportive, fair, respectful of personal values			
Would seek out further CEC in similar situations, would recommend CEC to others, educational value	Survey (Likert Scale “strongly disagree to “strongly agree”)	Healthcare providers and family members found the CEC helpful (92.3%, 87.0%); informative (81.1%, 88.0%); supportive (93.3%, 88.0); fair (92.9%, 84.3%); and respectful of personal values (92.4%, 85.1%). 73% of healthcare providers and 71.2% of family members did not find the CEC stressful Majority of clinical caregivers and family members would seek out further CEC in similar situations (95.2%, 80.4%); and recommend CEC to others (98.0%, 80.4%). Healthcare providers and family members strongly valued the problem-solving component of CEC. There were no statistical differences between healthcare providers and family members in beliefs concerning the educational value of CEC	[55]	
Usefulness	Helpfulness or satisfaction with process and decision	Mixed methods (Survey and open-ended questions/free text)	Almost all respondents stated they had an accurate understanding of the purpose of the HEC before the consultation began. Most staff members felt that the HEC was a valuable experience that would help them to some degree in their clinical practice. The two who felt it unhelpful were physicians. One stated each case is unique and would not have future applicability. Four of the six family members were very satisfied and two were very dissatisfied with the process. The two very dissatisfied family members were also the same individuals who disagreed with the Committee's opinion. Most staff members (23/32) were "very satisfied" with the process of the consultation. 7 were somewhat satisfied and 4 were somewhat dissatisfied. Of the 9 who were less than "very satisfied" only one disagreed with the final opinion of the HEC	[57]
Usefulness	Perceived benefit/utility of service	Mixed methods (Survey and open ended questions)	Quantitative: Q3. CECS involvement helped to (mark as many responses as appropriate): 68%—Enhance efforts to provide support to providers, patients and family 63.5%—Identify and verbalize moral concerns 63.5%—Facilitate solving real or potential problems 56%—Mediate between interests 51%—Strengthen decision-making processes 46%—Support providers, patient and family through hospital stresses and griefs 44%—Serve as an advocate for patient and family 23%—Interpret medical information given to patients and family Q4. Likert scale rating (1–5, Detrimental to Beneficial) of CECS overall effectiveness: 65%—5 (Beneficial) 22%—4 12%—3 (Neutral) 1%—2 0%—1 (Detrimental) Q7. Would welcome CECS participation in future: 94%—Yes 5%—Unsure 1%—No Qualitative: large data set, see study	[61]
Usefulness	Perceived benefit of service	Qualitative (open-ended questions)	Overall positive responses and helpfulness of service	[63]
Satisfaction	Satisfaction with service/future recommendation of CECS service	Survey	97% of respondents found the CECS deliberation to be at least somewhat helpful/very helpful 97% of respondents would recommend the service to colleagues	[66]
Usefulness	To what extent the MCD was regarded as useful	Mixed methods (Survey, open ended questions, interviews and focus groups)	*Organization of MCD* There was ample time/space in my working schedule for participating in MCD I was informed on MCD in time We have prepared this MCD meeting as a team *Content of MCD* I felt appealed to the case at hand in the MCD The discussion was relevant for our practice The way of discussing with one another was constructive Everyone had an equal share in the conversation In this MCD, I had enough opportunity to say what was on my mind It was good to analyse our reflections on the theme in an interrogative way *The MCD facilitator* The facilitator saw to it that everyone got his or her share during the MCD *Atmosphere during MCD* In the MCD, I could talk freely I felt safe during the MCD MCD: moral case deliberation Answers on 5-point Likert scale (1 1⁄4 totally disagree and 5 1⁄4 totally agree) Table 2. List of open questions addressed to all MCD participants How can this MCD be improved? What issues would you like to address in a future MCD? What has MCD brought to the team? What has MCD brought to you personally? What should, after this MCD, happen in practice? Do you have other general/supplemental remarks? MCD: moral case deliberation Mean score of caregivers (standard deviation within parentheses) (N 1⁄4 450)a 3.71 (s1.23) 4.24 (s1.17) 4.41 (s1.06) 2.46 (s1.46) 4.44 (s0.91) 4.48 (s0.84) 4.58 (s0.80) 4.51 (s0.78) 4.25 (s1.04) 4.47 (s0.96) 4.36 (s0.98) 4.53 (s0.78) 4.62 (s0.77) 4.62 (s0.77) 4.62 (s0.76)	[67]
Usefulness	Perceived helpfulness in patient care and physician education; indication of whether they would request future ethics consultation	Survey	86% respondents found consultation to be "very helpful" or helpful" in 1 or more aspects of patient care; 86% found it "very helpful" or "helpful" in 1 or more aspects of physician education; 97% would request an ethics consultation in the future	[68]
Usefulness	Perceived helpfulness in patient care and physician education; indication of whether they would request future ethics consultation	Survey	71% physicians stated consultation was "very important" in patient management, in clarifying ethical issues, or in learning about medical ethics; 96% would request ethics consultation in the future	[69]
Ability to Better Practice	Better handling of ethical challenges, better employee cooperation, better service quality, better relations to patients/users/next of kin, better work environment	Survey	41% to a large degree and 52% to some degree believed ethics activities led to better handling of ethical issues 24% to a large degree and 59% to some degree believed ethics activities led to better employee cooperation 24% to a large degree and 60% to some degree believed ethics activities led to better service quality 22% to a large degree and 54% to some degree believed ethics activities led to better relations to patients/users/ next of kin 21% to a large degree and 53% to a large degree believed ethics activities led to a better work environment	[72]
Usefulness	Attitudes regarding the overall helpfulness of the consult: helpful in the medical treatment of the patient; providing emotional support to patient/family; clarifying ethical issue; improving communication (patient/family and physician); improving communication (patient and family); mediating disputes; overall helpfulness	Survey	96% of physicians and 95% of nurses felt the consult was of at least some assistance, only 65% of patients or families thought the intervention was beneficial; 83% of physicians and 90% of nurses’ responses positively about the effects of the consult on medical management, while only 59% of patients or families saw medical benefit; 96% of physicians and 100% of nurses felt the consult was helpful in clarifying ethical issues, while only 65% of patients and families believed it was helpful	[74]
Usefulness	Would the respondent request an ethics consultation in the future	Survey	100% of respondent attending physicians would request an ethics consultation in the future	[77]
Usefulness	Was the ethics consultation helpful or detrimental to the family	Survey (Likert-scale)	70% agreement or strong agreement among family members that the CEC helped to identify (8/8), analyze (6/8), resolve (6/8), educate (6/8), was responsive to personal values (7/8), helped to educate others about ethical issues (6/8), was helpful (6/8), informative (6/8), supportive (7/8), and fair (6/8). Three of the 8 family members thought the CEC was stressful (40%). All but 1 physician agreed or strongly agreed with the positive process measures	[81]
Experience of Ethics Rounds	Helpfulness/perceived benefit of ethics rounds, role of philosopher/ethicist/moderator of rounds, improvement suggestions	Qualitative interviews	Improved personal ethical reflection (new perspectives, being more thoughtful, thinking about gray areas, etc.); feeling heard/not judged; however, not experiencing actual changes in daily work	[83]
Perceived Benefit	Reduction in ICU days and treatments in patients who did not survive hospitalization would be achieved through interventions (CEC?) that are viewed as beneficial by all involved parties: CEC assessed in terms of helpfulness, would seek CEC again, would recommend CEC to others; agreement with CEC recommendations	Survey (5-point Likert scale: strongly disagree, disagree, neutral, agree, strongly agree)	87% of nurses and physicians and patients/surrogates agreed or strongly agreed that CEC were helpful. More than 90% of nurses and physicians agreed or strongly agreed that they would seek them again and recommend them to others. Even though patients/surrogates found CEC somewhat more stressful than nurses/physicians, 80% agreed or strongly agreed that they would seek them again or recommend them to others. 13 surrogates disagreed or strongly disagreed with CEC recommendations, yet 7 would seek them again or recommend them to others	[82]
Usefulness	Extent to which the Department of Bioethics was found to be helpful by those who used their services	Survey (3-point Likert scale: (1) very helpful, (2) somewhat helpful, (3) not at all helpful)	46% of respondents had used the Department of Bioethics [responsible for ethics consultation]. 96% of those who called upon the DB found the experience to be either very helpful (64%) or somewhat helpful (32%). 25% of respondents had used the Ethics Committee. Of those who had, 97% found the experience to be either very helpful (53%) or somewhat helpful (44%). 92% of those who used the EC did so to assist a particular patient	[84]
Effectiveness	Effectiveness of the CES involvement in the case consultation	Survey (Likert-scale: beneficial, neutral, detrimental)	88% of ethicists and 83% of nurses reported ethics service involvement as "beneficial", whereas 65% of physicians reported as beneficial. Only a few respondents found it to be detrimental (3% of nurses, 4% of physicians)	[87]
Satisfaction	Satisfaction with intervention	Survey (5-item Likert scale)	Statistically significant differences in scores could not be shown from year to year. However, there are trends. In 1993 the lowest satisfaction scores (average 3.7) were given in the category of shared decision making. In 1994 and 1995 these scores increased (3.8 and 4.5) with efforts to address the low scores seen previously. In 1994 the lowest satisfaction scores were for increased knowledge of ethics issues with the consultation (average 3.3) and documentation adequacy (3.8)	[47]
Satisfaction	Positive experience, usefulness, would recommend/use again	Qualitative Interviews	Most clinicians found the consultation useful even those who had critical commentary, reporting an overall good experience. Many emphasized they would use the consultation service again or that it should be made more available/well known. Interviewers experience the case consultation positively, frequently due to the dilemmas being analyzed systematically and thoroughly. In consultations where there was no clear solution or advice, clinicians gave positive evaluations due to thoroughness of the discussion, and others reporting that the committee appreciated her own concerns and treated them seriously. Conclusion allowed clinicians to see the patient's wishes and values more clearly, gave the patient's relatives a feeling of being taken seriously, and that it was useful given its wider or more general impact not limited to the particular decision of the consultation having subsequent departmental impact	[62]
Overall Experience of Facilitator and Usefulness	Satisfaction/perception of facilitator and the experience 6 central qualities including (1) introduction and explanation, (2) ordering session, (3) listening and understanding, (4) critical reflection, (5) encouraging, (6) expertise The extent to which goals are met during the moral case deliberation	Survey (Likert-Scale)	(1) Introduction and Explanation = 7.8 (2) Ordering Session = 7.6 (3) Listening and Understanding = 7.9 (4) Critical Reflection = 7.5 (5) Encouraging = 7.6 (6) Expertise = 7.8 (1) to get knowledge of and insight in moral issues = 8.1 (2) to influence my attitude with respect to the case = 7.3 (3) to influence my behaviour with respect to the case = 7.1 (4) to improve my skills in dealing with moral issues = 7.5 (5) to deliver an answer or solution to the moral problem = 6.4 (6) to reach consensus within the group = 6.2 (7) to pay attention to reasons and arguments = 8.0 (8) to pay attention to feelings = 7.9 (9) to improve mutual understanding = 8.0 (10) to improve mutual cooperation = 7.9 (11) to active my job motivation = 9.7 (12) to frees my mind = 6.7 (13) to make me a better professional = 7.3 (14) to improve quality of care indirectly = 7.7 (15) to better ground decisions and reflect more on them = 7.7	[76]
Usefulness	Helpfulness of the consultation to the recipients	Survey (Likert-scale)	Over 90% of physicians or social workers agreed or strongly agreed that the CEC were helpful, informative, and supportive. Only 30% agreed or strongly agreed that the CEC was stressful. Of the 4 family interviews, half agreed or strongly agreed that the CEC was helpful and informative. 3 of 4 families strongly agreed the CEC was stressful. 2 of 4 families strongly disagreed that the CEC was supportive. Over 90% of physicians and social workers would recommend a CEC to others in similar circumstances. Only 2 of 4 families would recommend a CEC to others	[91]
Practical Implications	How did the participants appreciate the consultation, were the results helpful, were the consequences practical	Qualitative interviews	Team conflicts influenced consultation and implementation on the ward—during the consultation, conflicts were seen to be uncovered but not solved and moving forward were seen as essential to transferring results to wards. Overall, the hierarchical symmetry was seen as a barrier to development and implementation of solutions. Positively, ethics consultation can give impulse for changing communication within the team, and solutions directly relevant to a specific conflict lead to greater satisfaction with ethics consultation and CEC members especially when they reflect one's own opinion. Overall, while there is existing ambiguity following ethics consultation, with participants reporting that they remained unclear on the solution and that reporting instrument was insufficient to avoid misinterpretation and communication, most participants felt discussion was useful for solving ethical conflict, revealing underlying team conflict, and to contact the CEC in case of further ethical conflict	[80]
Usefulness	Was the ethics consultation helpful/was it helpful or detrimental to the family	Survey (Likert-scale)	Very Helpful = 23 Somewhat Helpful = 9 Neither Helpful Nor Detrimental = 21 Somewhat Detrimental = 0 Very Detrimental = 2 No Response = 1	[78]
Satisfaction	Providers’ impression of the intervention	Survey (5-item Likert scale)	Large data set. Refer to study	[89]
Satisfaction	Overall satisfaction with hospital experience	Survey (5-item Likert scale)	Respondents in both arms had generally positive perceptions with no significant difference between them Intervention 86.1% nurses and physicians reported that patients were "somewhat satisfied" or "very satisfied" compared to 74/8% of patients/surrogates the same Control arm, figures for the same were 81.4% and 83.6% Nurses and physicians in the intervention arm report that 65.6% of patients had "little suffering" or were "free of suffering" with 52.6% of patients/surrogates reporting the same Control arm: 58.9% and 57.9% respectively	[48]
Satisfaction and Usefulness and Experience with Patient/Next of Kin Involvement in Consultation	Clinician satisfaction with consultation Clinicians’ reasons provided for requests (broad discussion, better equipped for future situations, advice, external perspective, support, learning, clarifying values, disagreement among professionals, disagreement with family/next of kin/patient, improve cooperation) and the perceived usefulness of the consultation in that respect Characterization and importance/usefulness of patient/next of kin involvement in consultation process (positive, unproblematic, new/important information, problematic, difficulty clarifying, conflicts)	Survey (Likert scale) Survey (Likert scale) Survey (tick-boxes)	Mean Likert score per question: (1) meeting stakeholders with respect = 4.96 (2) overall positive experience = 4.82 (3) felt listened to = 4.81 (4) received sufficient information = 4.68 (5) would recommend CEC = 4.85 (6) was allowed to make important contributions = 4.77 (7) learning about ethical dilemmas = 4.44 (8) increased knowledge in navigating ethical conflict = 4.33 (9) overall new information = 3.98 (10) changes in opinion = 2.46 Proportion who disagree somewhat/strongly: (1) meeting stakeholders with respect = 0/53 (2) overall positive experience = 1/51 (3) felt listened to = 0/53 (4) received sufficient information = 1/53 (5) would recommend CEC = 2/53 (6) was allowed to make important contributions = 1/53 (7) learning about ethical dilemmas = 3/52 (8) increased knowledge in navigating ethical conflict = 1/52 (9) overall new information = 6/52 (10) changes in opinion = 21/52 CEC mean Likert score: (1) meeting stakeholders with respect = 4.90 (2) overall positive experience = 4.64 (3) felt listened to = 4.89 (4) received sufficient information = 4.68 Indicated by proportion of clinicians (% (N)): (1) broadening discussions = 93% (42) (2) better equipping for future similar situations = 67% (30) (3) getting an external perspective = 62% (28) (4) getting advice about a decision = 64% (29) (5) getting support for decisions = 60% (27) (6) learn from a difficult case = 58% (26) (7) clarifying values = 44% (20) (8) disagreements among professionals = 31% (14) (9) disagreements between professionals: 27% (12) (9) improving cooperation = 22% (10) Average score for usefulness: (1) broadening discussions = 4.50 (2) better equipping for future similar situations = 4.40 (3) getting an external perspective = 4.69 (4) getting advice about a decision = 4.32 (5) getting support for decisions = 4.78 (6) learn from a difficult case = 4.40 (7) clarifying values = 4.70 (8) disagreements among professionals = 3.50 (9) disagreements between professionals: 4.33 (9) improving cooperation = 4.00 CEC Respondents (N = 16) (1) Positive = 15 (2) Unproblematic = 10 (3) New and important information was revealed = 6 (4) Problematic because it was difficult to speak freely = 1 (5) Difficult to clarify medical/professional information well enough = 1 (6) Conflicts inhibited the ethical discussion = 2 Clinicians (N = 17) (1) Positive = 14 (2) Unproblematic = 5 (3) New and important information was revealed = 3 (4) Problematic because it was difficult to speak freely = 3 (5) Difficult to clarify medical/professional information well enough = 2 (6) Conflicts inhibited the ethical discussion = 0	[73]
Satisfaction	Degree to which service meets expectations and need (themes described: responsiveness, willingness to consult, institutional role, and areas for improvement)	Qualitative interviews	**Responsiveness of the Ethics Consultant** Participants felt that ethics consultant was respectful, responsive, accessible, and approachable. Amount of time spent on consult was sufficient to help address the ethical concerns **Willingness to Consult** Willing to consult ethics service again; ethics service fills a gap in case and knowledge that otherwise wouldn't be filled. Differed on timing of *when* they would consult service again. Most participants comfortable with the outcome; even when medical outcome poor, service seen to empower clinical and ethical decision-making, promote patient safety, honour patient wishes, and facilitate team communication and cohesion **Institutional Role** Participants held varying opinions. Respondents mentioned ES had a role in advocating for patients and connecting team to institutional resources, while others commented on interface between legal and regulatory system offered by ethics service. One commented on bad reputation of ethics service as implying "bad behaviour" or as a means of "policing healthcare professionals' decisions" **Identifying Areas for Improvement** Most did not have recommendations; however, comments were made about making ethics service better known. Other suggestions involved involving members of the care team during discussions	[94]
Experience of the MCD	Promoting carefully considered decisions, giving a better explanation and justification for some decisions, which leads to quality	Qualitative interviews and focus groups	Participants reported the MCD promoted carefully considered decisions, giving a better explanation and justification for some decisions, which leads to quality	[93]

Among the 16 studies evaluating usefulness, eight reported CEC to be useful. These eight studies understood usefulness as resolving issues or conflict (n = 5), identifying, analyzing, and clarifying issues and/or values (n = 5), and in providing education (n = 3). Some studies concluded that the intervention improved interpersonal and professional qualities such as mutual understanding and cooperation, personal ethical reflection and insight, and ability and confidence to act in practice. Respondents in a few studies would seek CEC again (n = 2) or would recommend the service to others (n = 4). Insights and cross-comparisons between participant groups who experienced higher or lower levels of usefulness are difficult to ascertain given insufficient statistical power or incomplete reporting on the perspectives of certain stakeholder groups, such as patients and families (Table 5).

Respondents in almost all studies reported a positive or beneficial encounter with CEC either in terms of overall experience, quality, usefulness, value, helpfulness, or satisfaction (n = 28), with healthcare professionals being the most frequently examined group (n = 26).^3^ Seven studies reported a moderate to high level of satisfaction with the CEC experience. These were described in various ways: an inclination to use the service again or recommend the service to others, the degree of consensus between stakeholders, the ability to meet perceived goals, general satisfaction with the service or intervention, the positive or negative nature of the experience, practical usefulness, and the impression of the intervention. White, Dunn, and Homer reported specifically on low levels of satisfaction, with shared decision making, documentation adequacy, and increased knowledge of ethical issues receiving the lowest satisfaction scores [47]. Only one study found respondents’ overall experience with CEC was negative [49], although other studies suggested areas for improvements [83]. Negative experiences were attributed to factors such as a lack of structure in the consult, a lack of timeliness, or an absence of integration into clinical practice [49, 83].

#### Process factors

Process factors comprised the second most reported outcome domain (n = 23). Process factors referred to any set of activities that occurred between the CEC provider and other stakeholders, such as identifying, clarifying, resolving ethical issues, reaching consensus, and facilitating understanding of different viewpoints (Table 6). Process factors were identified by qualitative interviews or focus groups (n = 13), survey (n = 10), mixed methods (n = 3) and record review (n = 1).

**Table 6:** Process factors reporting

Process factors assessed (name construct)	Outcome description	Outcome measure	Results	Reference #
Reasoning for Accessing CEC	For what purposes do the participants contact the CEC	Qualitative Interviews	Doctors report contacting CEC in order to receive moral backing for decisions that were made, obtaining an elucidation of the ethical aspects of the case, to receive concrete advice, and to have the case discussed by people that are not directly involved in the case, or a combination of one or more of these reasons	[49]
Consolidating Care and Clarifying Perspectives	Striving for common care goals and creating a shared vision of care in the specific case Involved several sub-processes: deliberating ethics (ethical reflection); unifying interactions (increased interprofessional understanding and agreement, providing basis for decision making and common care objectives, increasing shared understandings leading to unity within the team); group strengthening (contributing to atmosphere of mutual understanding/acknowledgement); decision grounding (decision grounded in multidisciplinary perspective)	Qualitative Interviews	Core category of consolidating care Consolidating care—striving for common care goals and creating a shared view of care in the specific case. Consolidating care by clarifying perspectives. This involved multiple processes: a) deliberating ethics (raising and clarifying ethical values and relating them to possible courses of action), b) unifying interactions (increasing interprofessional understanding and agreement, providing a basis for decision making and common care objectives; increased shared understandings contributing atmosphere of mutual understanding/acknowledgement) C) group strengthening—contributing atmosphere of mutual understanding/acknowledgement; d) decision grounding—decision grounded in multi-disciplinary perspective Core category supported by related process of clarifying perspectives	[50]
Support	Support regarding interpersonal relationships and interactions among patients, family, nurses, and physicians	Survey	10/13 nurses (13 who responded to this question) (77%) felt that the most important aspect of CEC involved support regarding interpersonal relationships and interactions among patient, family, nurses, and physicians	[51]
Facilitating Advanced Communication (Helpfulness)	Helpfulness in identifying, analyzing, and resolving ethical issues, helpful in educating all parties, and in helping parties present their personal views	Survey (Likert scale)	Healthcare providers and family members found the CEC to be helpful in identifying the ethical issue (87.7%, 86.7%); analyzing ethical issues (86.5%, 84.6%); and resolving ethical issues (73.9%, 71.2%); helping to educate all parties (80.0%, 81.9%); and helping parties present their personal point of view (80.9%, 84.5%)	[55]
Moving It Along (Advancing Care)	Transitioning the patient out of the hospital setting—can be constrained if any decision for care or implementation of care is not morally acceptable. In volved 3 stages: (1) moral questioning; (2) seeing the big picture; (3) coming together	Qualitative Interviews	Core category of Moving it Along, relates to transitioning care and the tension created when there is a moral issue that impedes progress of the patient in the system. 3 stages: (1) Moral questioning—identifies reasons for calling the ethics consultation service (i.e. varied perceptions, asking questions, calling the question); (2) Seeing the big picture—comprised of two sub-themes: opening to new ideas and consensus-building; (3) Coming together—realistic and ethically acceptable resolution was achieved through (a) working on the same page—variety of ethics interventions employed such as interview, consultation write-up, and meeting with stakeholders, professionals valued ethics consultations because of the improved communication and respect. They believed their questions were heard and that their point of view was taken seriously, education and new information about decision making principles were valued. (b) resolving and reflecting—some professionals expressed dissatisfaction when expectations were not met; for e.g., expectation to have a solution and clear specific plan	[56]
Consensus Regarding Care Achieved	Whether goal of HCEC was achieved (for HCEC group: if any of the morally acceptable options suggested by the individual ethics consultant was followed; for UC group: if patients/family members and health care team members agreed on options for goals of medical care)	Record review (Review of Medical Record/HCEC Record)	85% of patients in HCEC group reached consensus re: goal of medical care after HCEC service vs 24% in UC group reached consensus** Considering crossover, 35 pts who received HCEC service more likely to reach consensus than 27 pts who didn't	[65]
Impact of MCD	Individual participants’ experience of MCD and results of MCD for Care Practice	Qualitative interviews and focus groups	Robust set of qualitative data reported. See study for data set	[67]
Integrating Familial Perspectives	Bringing together elements considered by family as crucial to the end-of-life decision making process; active listening, asking the right questions so that families could bring to the fore what really mattered to the patient and family, and exploring possible options	Qualitative Interviews	"Of the participants who did have access to a clinical ethicist during the process of decision-making, the value of the ethicist was clearly stated":—illuminating important and relevant questions that no one else was asking (asking the right questions); listening to families (active listening); exploring possible options	[71]
Evaluation of CEC Service (Impact)	Rating by attending physician of consultation's importance in clarifying ethical issue, educating team, increasing their confidence in patient management, and in making patient management decisions	Survey	Physicians reported finding consultation to be either "very" or "somewhat" important in clarifying ethical issues (95%), educating the team (100%), increasing confidence (93%), and managing patients (95%)	[77]
Identification of Ethical Issues	Did consultants identify issues that requesters had not recognized	Mixed methods (Survey and Medical Chart Review	Respondents said 14 consultations identified ethical issues that requesters had not recognized; medical charts partially substantiated physician responses. Physicians demonstrated high recognition of ethical issues involving providing or withholding life support. Physicians demonstrated low recognition of issues involving proxy decision making for incompetent adults and issues concerning terminal care	[79]
Process Hypotheses (Impact)	Process hypotheses are consistent with the goals of the consortium's consensus statement and serve an important role in ethics in ICUs in helping to a) identify ethical issues; b) analyze ethical issues; c) resolve ethical issues; d) educate about ethical issues; e) help present personal views	Survey (Likert scale)	70% agreement or strong agreement among family members that the CEC helped to identify (8/8), analyze (6/8), resolve (6/8), educate (6/8), was responsive to personal values (7/8), helped to educate others about ethical issues (6/8), was helpful (6/8), informative (6/8), supportive (7/8), and fair (6/8). Three of the 8 family members thought the CEC was stressful (40%). All but 1 physician agreed or strongly agreed with the positive process measures	[81]
Process (Impact)	Subjective evaluations of CEC process: respectful of values, CEC helped in identifying, analyzing, resolving, educating, presenting view	Survey (5-point Likert scale: strongly disagree, disagree, neutral, agree, strongly agree)	87% of nurses and physicians and patients/surrogates agreed or strongly agreed that CEC were helpful. More than 90% of nurses and physicians agreed or strongly agreed that they would seek them again and recommend them to others. Even though patients/surrogates found CEC somewhat more stressful than nurses/physicians, 80% agreed or strongly agreed that they would seek them again or recommend them to others. 13 surrogates disagree or strongly disagreed with CEC recommendations, yet 7 would seek them again or recommend them to others	[82]
Helpfulness/Positive impact in their perceived role	Perceptions of clinical ethicist’s greatest positive impact on the case	Survey	43% of ethicists perceive their greatest positive impact in consultation is to identify or clarify key issues and options in care, followed by assisting in finalizing end of life care plan (15%) and support participants (14%). 25% of nurses believed the ethicist's greatest positive impact was to assist in finalizing end of life care plan (25%), followed by allow participants to voice/discuss (21%) and identify or clarify key issues and options in care (25%). 22% of physicians identified allow participants to voice/discuss, followed by identify or clarify key issues and options in care (17%), and then explain legal issues (13%) and support participants (13%). Only 4% of physicians identified "assist in finalizing end of life care plan", contrary to ethicists and nurses	[87]
Purpose	What reasons were the cases brought to the committee and what role does the committee specifically fulfill in the case	Qualitative interviews	Conflicts between professionals regarding solutions was embedded in many cases but not explicitly stated as a motivation. All cases were submitted with the purpose of getting decision-making support in decisions where ethical challenges were salient. the most common reason for requesting was to obtain a systematic and comprehensive analysis of a difficult ethical dilemma from a more distant position outside the team. Among other reasons cited were helping clinicians deal with fundamental questions of a problem, getting a critical evaluation, getting specific advice in cases involving life and death decisions, creating an opportunity to share responsibility with others, getting moral support in a non treatment decision when treatment was medically indicated,	[62]
Perceived Helpfulness (Impact/Purpose)	In what ways was the ethics consultation helpful to families and what did the consultation do to help the families	Qualitative interviews	Content analysis of interviews yielded that ethics consultations are helpful to patients and families in the following domains: clinical clarity, moral or legal clarity, motivation, facilitation, implementation, interpretation, consolation and support	[78]
Expectations (Impact/Purpose) And Overall Assessment of CEC and impact on values	Respondent’s expectations of the CEC	Mixed methods (Survey—yes/no, and optional free text response) Survey (11 items adapted from a tool developed by White, Dunn and Homer [47] and outcomes measures for EC (ASBH 2011)	Top 6 expectations of CEC were: facilitate communication between team and pt/family, clarify/define a plan of care, provide a neutral perspective, provide information, facilitate communication among team members, and provide a safe space Overall assessment of ECS was favourable. More than 90% felt the consultant explained things well, more than 80% felt the consultation validated the team's approach and provided support, and more than 70% felt the ECS clarified uncertainty, gave them a better understanding of ethical issues, and helped resolve a patient care problem. More than 80% felt the CEC recommendations were consistent with the organization's values, respected the respondent's values, and were consistent with their personal values. More than 60% felt the CEC helped clarify the values of the patient and/or patient's family, and helped respondents clarify their own values. Qualitative interviews uncovered some comments suggesting the EC could have communicated more effectively with members of the health care team	[90]
Importance of EC	Importance of EC to participants	Survey (Likert scale)	Over 90% of physicians and social workers agreed or strongly agreed that the ethics consultant was important in identifying and analyzing ethical issues. 70% agreed or strongly agreed that the consultant was important in resolving ethical issues, 74% agreed or strongly agreed that the EC was important in increasing confidence in patient management. Only 2 of 4 family members agreed the EC was important for identifying and analyzing ethical issues, educating the family, and increasing confidence in patient management. 3 of 4 families strongly disagreed that the EC was important for resolving ethical issues. Interviews with physicians and social workers express positivity with the CEC, and appreciated the help and hand-holding, another said it was good at delineating issue sand allowed for different views to be voiced	[91]
Process of Deliberation (Harvest) (Impact/Purpose)	Authors use the term "harvest" to identify nurses' reporting of MCD impact on professional practice. "Harvest" includes cooperation; developing a critical attitude towards practice; empowerment and enhancement; understanding; boundaries, limitations and self-care; quality of care; and exploring policy, paradigms and vision	Qualitative interviews	Intervention revealed team conflicts, but did not solve them. Existing concerns regarding solutions were not addressed given that participants were afraid of hierarchical structure on wards and in departments, further hindering implementation of solutions. Solutions can be too complex or existing ambiguity in terms of solutions can remain. Disappointment was expressed because consultation was marred by communication barriers. Record was a sufficient instrument to communicate the solution. Though some participants had questions at the end of consultation, most participants felt discussion was useful to solve the ethical conflict, reveal the underlying team conflict, and to contact the CEC in case of further ethical conflict	[86]
Program Impact	What is the role/purpose of the EC in relation to the perceived ethical issues	Mixed methods (Medical record/case note review and qualitative interview)	EC is described as preventative—assess family ability to be a partner in case, identifies barriers to decision-making, builds trust between the medical team and family, and sets expectations about ECMO early on. All 30 physicians regard early EC protocol positively and note that without the protocol, ethics would be consulted only when conflict is intractable. Bedside nurses, APPs, and fellows recognize additional benefit of establishing EC as a routine being that involving ethics is not seen as a failure or action of a whistleblower	[88]
Awareness of Others Perspectives and Interests and Allowing Deeper Exploration and Insight and Enhanced Collaboration and Concrete Results	Participants voiced appreciation that all MCD participants were encouraged to contribute and that disagreements were discussed in less polarizing ways MCD enabled teams to explore in great detail how they handled a given protocol and why they had done so. Participants appreciated the time MCD offers for talking about cases, restoring participants' sensitivity to the particularity of the case at hand This domain included 5 items: (1) Greater opportunity for everyone to have their say; (2) better mutual understanding of each other’s reasoning and acting; (3) enhances mutual respect amongst co-workers; (4) I and my co-workers manage disagreements more constructively; (5) more open communication among co-workers This domain contains 3 items: (1) Find more courses of action in order to manage the ethically difficult situation; (2) consensus is gained amongst co-workers in how to manage ethically difficult situations; (3) enables me and my co-workers to decide on concrete actions in order to manage ethically difficult situations	Qualitative interviews and focus groups Qualitative interviews and focus groups Survey (Euro MCD Instrument) Survey (Euro MCD Instrument)	Participants voiced appreciation that all MCD participants were encouraged to contribute and that disagreements were discussed in less polarizing ways MCD enabled teams to explore in great detail how they handled a given protocol and why they had done so. Participants appreciated the time MCD offers for talking about cases, restoring participants' sensitivity to the particularity of the case at hand Most frequently experienced outcomes during MCD session (t1, n = 22): "better mutual understanding of reasoning and behavior (84%)", "more open communication among co-workers (82%)". Most frequently experienced outcomes in daily work after MCD sessions (t1, n = 22): "enhances mutual respect among co-workers (55%) Least often experienced outcomes in daily work after MCD sessions: "enables me and my co-workers to decide on concrete steps to manage ethically difficult situations (75%)"	[93]
Impact on Practice	First line managers’ experiences of what Moral Case Deliberation meant for daily practice	Qualitative interviews	Managers experienced an enhanced ethical climate including a closer knit and more emotionally mature team and morally strengthened individuals. Their experience was ethics leaving its mark on everyday work, and more morally grounded actions. Despite perceptions of organizational barriers, managers felt inspired to continue ethics work	[92]
Value	Moral space that was created, allowing for reflection, analysis, negotiation, and processing of ethical problems (intrapersonal and interpersonal worth of ethics consultation)	Qualitative interviews	**Intrapersonal Worth** Ethics service created a moral space that allowed participants to respond to the emotional, cognitive, and behavioural demands presented by ethical problem **Interpersonal Worth** Consults allowed participants to respond to other parties involved in the ethical question including patients, family members, and team members	[94]

The most reported subtheme in the process factors domain was purpose and/or impact of the intervention (n = 14). Other subthemes included establishing consensus and integration (n = 3), helpfulness (n = 2), identification (n = 1), advancing care (n = 1), support (n = 1), and clarification (n = 1).

With respect to the dominant subdomain, the data demonstrate diverse understandings within and across stakeholder groups regarding the purpose and impact of ethics intervention. Many study respondents perceived ethics involvement to be most effective with respect to identifying or further elucidating core [ethical] tensions or barriers to resolution (n = 8), achieving resolution, consensus, or clarity on the problem, plan of action, or desired outcome (n = 7), and/or facilitating the sharing of viewpoints and better communication between stakeholders (n = 5). Five studies additionally reported ethics intervention as having an impact by improving individuals’ feelings of safety and support (n = 2), trust (n = 2), and confidence in decisions (n = 1). Two studies reported ethics intervention as increasing mutual understanding and respect within and between stakeholder groups, as well as providing valuable education (n = 4) or an objective opinion on the problem at hand (n = 3).

Some studies, however, were less sanguine about these descriptors. For example, Weidema et al. reported that ethics intervention revealed team conflicts, but maintained that it did not assist in solving them due to the ethical issues being too complex or ambiguous [86]. This finding is corroborated by Vrouenraets et al. who reported that enabling concrete steps to navigate ethically difficult situations was the least common outcome associated with ethics interventions [93]. In some studies, the positive impact of CEC was not consistent across stakeholder groups. Yen and Schneiderman, and Schneiderman et al., for example, found that the positive experience reported by physicians was not experienced by family members, with 75% strongly disagreeing that an ethics intervention was important for resolving ethical issues [81, 91]. Other studies reported dissatisfaction when expected outcomes were not met, such as the expectation of resolution or the formulation of a clear plan of action [56]. In studies that provided recommendations for process-related improvement, respondents prescribed better communication with the health care team [90]. Poor communication, complexity, and ambiguity were also reported as barriers to the resolution of ethical problems in other studies in the process domain category [86].

#### Clinical factors

Nineteen studies were coded under the clinical factors domain, which related to a change in the clinical care of the patient, including, but not limited to, adherence with a recommendation, agreement with a recommendation, a decision about care or treatment, concordance between patient wishes and treatment delivered, a change in treatment plan, a reduction of non-beneficial treatment, conflict resolution regarding patient care, and patient survival/mortality (Table 7). The majority of studies in this domain utilized record review as the method of data collection (n = 15). Other methods included survey (n = 6), qualitative interviews (n = 2) and mixed methods (n = 1).

**Table 7:** Clinical factors reporting

Clinical factors assessed (name construct)	Outcome description	Outcome measure	Results	Reference #
Nonbeneficial treatment and Mortality and Suffering	Patients receiving ventilation and/or receiving artificial nutrition and hydration between intervention and control Died in Hospital Amount of perceived patient suffering from provider perspective, patient perspective, and/or surrogate perspective	Record review (Medical record review) Record review (Medical record review) Qualitative interviews (“daily interviews”)	Receiving ventilation (median days): Intervention (N = 56): N = 15 Control (N = 52): N = 14 Receiving artificial nutrition and hydration (median days): Intervention (N = 56): N = 17 Control (N = 52) l: N = 16 Intervention (N = 174): N = 56 (32.2) Control (N = 210): N = 52 (24.8) Nurses/Physicians in the intervention arm reported 65.6% had "little suffering" or were "free of suffering with 52.6% of patients/surrogates reporting the same. In the control arm, the figures were 58.9% and 57.9% respectively	[48]
Impact of CEC on the decision	Agreement with decision, treatment plan changed significantly after CEC	Survey (Likert scale)	Majority of clinical caregivers and family members agreed with the decision reached in the CEC (81.3%, 71.8%); both healthcare providers and patient/ family members perceived similar degrees of changes in the treatment plan following CEC	[55]
Quality of communication index	Presence of advance directive, DNR order, orders to withhold/withdraw life-sustaining treatment, limits of care, consultations requested for pastoral care, social services, pain management	Record review (Patient chart review (observational tool developed to gather documentation from medical records—score of "1" given each time an entry appeared in the record)	Ethics proactive group had significantly higher communication scores than other 2 groups; patients who died had significantly higher communication scores than those discharged alive; significant diff in proactive ethics group compared to baseline/control in having DNR and other life-sustaining tx decisions made in course of ICU care, decisions to withhold/withdraw life-sustaining treatments (communication & decision-making)	[60]
Consultant response to requests	Consultant suggestions for changes in treatments and orders	Record review (Consultant records)	Consultant suggested changing/discontinuing orders in 48/104 cases	[68]
Consultant response to requests	Consultant suggestions for changes in treatments and orders	Record review (Consultant records)	Consultant made specific recommendations in 48/51 cases; DNR order recommended in 13 cases and written in 12/13 cases	[69]
Consensus and Implementation Rate	Whether there was consensus amongst CEC participants regarding recommendation Whether CEC recommendation was implemented	Record review (Medical chart review (each CEC is documented using semi-structured protocol for patient record)	High mutual consensus rate amongst all participants in CECs (90.8–96.5%); no significant difference btwn patient groups (ICU, LCU, PCU aka high consensus for all groups) High rates of implementation/adherence to final CEC recommendation (89.7–100%); no significant difference btwn patient groups (ICU, LCU, PCU aka high implementation rate for all groups)	[70]
Less Use of Coercion	Less use of coercion	Survey	13% to a large degree and 26% to some degree believed ethics activities led to less use of coercion. 51 respondents formulated additional outcomes. The four most prevalent were: heightened awareness of ethical challenges, a lower threshold for raising and discussing ethical challenges among colleagues, an increased concern with patient/user needs and interests, and increased knowledge (i.e., of ethics, law, clinical practice)	[72]
Adherence to CEC Recommendations	Whether the CEC recommendations were followed	Record review (Retrospective chart review)	In all cases the recommendations of the consultants were followed with the exception of 1 patient who died before the consult could be completed. However, in some instances, delay occurred in the implementation of recommendations	[74]
Change in Patient Management	Whether the consultation resulted in a change in patient management	Survey (Yes/No question)	(8) to pay attention to feelings = 7.9 (9) to improve mutual understanding = 8.0 (10) to improve mutual cooperation = 7.9 (11) to active my job motivation = 9.7 (12) to frees my mind = 6.7 (13) to make me a better professional = 7.3 (14) to improve quality of care indirectly = 7.7 (15) to better ground decisions and reflect more on them = 7	[77]
Change in patient management	Did the consultation change patient management	Mixed methods (Survey and medical chart review)	Respondents said 18 consultations changed patient management considerably, 16 changed management slightly, and 10 did not change patient management. The chart review demonstrated that most management changes occurred because consultation persuaded physicians to withhold life support therapies that physicians had planned to use. However, 7 consultations persuaded physicians to give life support therapies they had not planned to use. Consultations changed management in at least half of cases involving questions of adults' competence to refuse therapy or of proxy decision making for incompetent adults. Consultations sometimes persuaded physicians to override parents' medical decisions for their children when those decisions appeared contrary to the child's presumed interests. Few consultations changed the use of laboratory tests or limb restraints for terminal patients	[79]
Nonbeneficial treatment	Number of days in the ICU and life-sustaining treatments in patients who died before discharge	Record review (Medical record data)	In those pts who died before discharge, there was a reduction in ICU days (p = .03), days receiving artificial nutrition/hydration (p = .05), percent on ventilation (p = .08), and days receiving ventilation (p = .05) in pts receiving an ethics consultation compared with control pts	[81]
Nonbeneficial treatment and	ICU days, hospital days, and life-sustaining treatments in those patients who did not survive to hospital discharge (because these represent a failure to achieve a fundamental goal of medicine the authors called them "nonbeneficial treatment")	Record review (Medical record data (prior to and after entry to the study)	Among those patients who received the intervention (n = 173), compared with control pts (n = 156) but did not survive to discharge from hospital, hospital days (P = .01), days spent in the ICU (P = .03), and days receiving ventilation (P = .03) were reduced. Days receiving artificial nutrition/hydration (P > .50 for all outcomes) showed no significant differences between groups. A pattern towards reduction of hospital and ICU days associated with CEC pts vs UC was observed at all the hospitals	
Mortality	Hypothesized that CEC would not increase mortality relative to UC	Record review (Medical record data)	No significant difference in mortality rate between those patients who received CEC and those who did not	[82]
Agreement between the ICU team and patients/surrogates and Provision of palliative and chaplaincy services	Agreement between the ICU team and patients/surrogates on goals of care (i.e. to limit life-sustaining interventions, withdraw life-sustaining treatments), and agreement reached on various other conflicts Provision of palliative and chaplaincy services	Record review (Pre-post CEC chart review of DNR orders entered, and [presumably] medical record data [unspecified which data relates to measuring agreement]) Record review (Pre-post CEC chart review of palliative care and chaplaincy consultations, recommendations for these services by the ethics consultants)	After CEC all 17 patients in the WOLST (withdrawal of life sustaining therapy) group reached agreement to withdraw life sustaining therapies. 5 had DNR orders prior to CEC and 12 additional patients had DNR orders after CEC. Among 25 patients in the LOLST (limitation of life sustaining therapy) group, agreement was achieved to limit life sustaining therapy for 19 patients. Of these patients, 6 already had a DNR order prior to CEC; DNR orders were entered for an additional 13 patients post CEC. In the "other" group (various ethical issues group), agreement between parties was achieved in 9 out of 11 cases; following CEC 3 additional patients had a DNR order entered After CEC, recommendations were made for an additional 8 (15%) palliative care and 9 (17%) chaplaincy consultation	[85]
Change in patient management	Effect of CEC on patient management	Survey	Statistically significant differences in scores could not be shown from year to year. However, there are trends. In 1993 the lowest satisfaction scores (average 3.7) were given in the category of shared decision making. In 1994 and 1995 these scores increased (3.8 and 4.5) with efforts to address the low scores seen previously. In 1994 the lowest satisfaction scores were for increased knowledge of ethics issues with the consultation (average 3.3) and documentation adequacy (3.8)	[47]
Quality of Care	Improvement in quality of patient care	Record review (Patient medical records)	Robust data. See study	[89]
Patient related outcomes	Related to the plan of care	Survey (yes/no item)	Only 32% of respondents indicated the patient's plan of care changed as a result of the CEC	[90]
Patient treatment changes	Treatment changes as a result of EC	Record review (Medical record data)	Robust data. See study	[91]
Changes in Treatment Decisions and Improvement on organizational level	Reported change in treatment plans as a result of the MCD This domain includes 3 items: (1) I and my co-workers become more aware of recurring ethically difficult situations; (2) contributes to the development of practices/policies in the workplace; (3) I and my co-workers examine more critically the existing practices/policies in the workplace/organization	Qualitative interviews Survey (Euro MCD instrument)	Reported change in treatment plans as a result of the MCD	[93]
Patient Status at Discharge	Patients’ status at hospital discharge	Record review (Medical Record Review/HCEC Record Review)	79% pts in HCEC group and 72% pts in UC died at hospital discharge (p = .56)	[65]

Studies that reported on clinical factors examined a narrower set of characteristics compared to other domains; patient management and provision of care was the most frequently reported construct (n = 11). Patient management and provision of care included the following indicators: the presence of, or recommendation to change, particular orders or decisions (e.g. do-not-resuscitate orders, withholding/withdrawal orders, life-sustaining treatment, limits of care, requests for services, and pain management), provision of palliative care and/or chaplaincy services, agreement with a decision, and a change in treatment plan, plan of care, or patient management. Other subthemes reported in this domain included non-beneficial care (n = 3), patient mortality (n = 3), clinical consensus (n = 2), suffering (n = 1), quality of care (n = 1), and coercion (n = 1).

Constructs, outcomes, and measures varied considerably between the studies examining patient management and provision of care, despite their similarities. For example, Dowdy, Robertson, and Bander measured the presence of particular constituents of care, understood by the term *quality of communication index—*communication and decision making that resulted in a treatment order between a control and ethics intervention group. These researchers noted a “higher communication score” and significant difference between intervention and control with respect to the presence of do-not-resuscitate (DNR) and life-sustaining treatment decisions [60]. Another study by Cohn et al. examining communication and decision-making, measured the level of agreement with the decision and the degree of change that occurred due to CEC. Healthcare provider respondents were significantly more likely than family members to report a high degree of agreement with the outcome of the ethics consultation, and both healthcare providers and patient/family perceived similar degrees of change to the plan of care following CEC [55]. Other studies measuring patient management considered whether consultations changed patient care, measuring the change rather than the presence (or not) of particular types of care. Outcomes demonstrated that the majority of respondents reported CEC as changing patient management in some regard, with most changes occurring due to the consultation prompting the withholding of life support therapies that would have otherwise been used [79]. Thus, despite parallels across the various constructs, differences exist with regards to the terminology (and their related understandings) and measurement of outcomes.

Wide variation also existed in measures and outcomes with respect to non-beneficial treatment, with minimal consensus on what might be considered and measured as non-beneficial, as well as what can be concluded with respect to the impact of CEC on the provision of non-beneficial treatment. For example, Schneiderman, Gilmer, and Teetzel measured the number of ICU days and life-sustaining treatments in patients who died before discharge. They reported a reduction in ICU days, days receiving artificial nutrition and hydration, percentage on ventilation, and days receiving ventilation in patients receiving consultation compared with the control [81]. In later research, which focused on number of ICU days, hospital days, and life-sustaining treatment in patients who did not survive to discharge, Schneiderman et al. found no significant difference between intervention and control; however, a pattern in the reduction of hospital and ICU days with ethics intervention was observed. Additionally, this study justified outcomes as “non-beneficial” because they were perceived to “represent a failure to achieve a fundamental goal of medicine” [82]. Another study by Andereck et al. examined non-beneficial treatment among patients receiving ventilation and/or artificial nutrition. While the intervention and control groups varied by one median day, the authors did not make any causal claim with respect to ethics intervention and reduction in the provision of these treatments [48]. With respect to patient outcomes, no significant difference in mortality rate was observed between participants who received ethics intervention and those who did not [48, 65, 82].

#### Personal factors

Personal factors was the fourth most reported on domain (n = 8). Studies coded under this domain related to changes in personal state or stakeholder (patient/family/surrogate/health care professionals) perspective or experience; for example, moral distress, enhanced knowledge, and/or feeling supported (Table 8). Data collection methods included survey (n = 7) and qualitative interviews (n = 1).

**Table 8:** Personal factors reporting

Personal factors assessed (name construct)	Outcome description	Outcome measure	Results	Reference #
Moral Distress	Staff moral distress levels	Survey	28/35 respondents reported that involving the clinical ethics service at least somewhat reduced their own moral distress; 7/35 reported no decrease	[66]
Clarity and Physician Confidence and Physician learning	Did consultations help to clarify thinking about ethical issues Did consultations increase physicians’ confidence in their final management plans How much did the consultations teach physicians?	Survey	39 consultations clarified thinking about ethical issues 41 consultations increased requesters' confidence in their final management plans, and only 3 decreased their confidence Physicians reported learning much from 42 consultations (98%)	[79]
Perceived value	How participants valued the importance of the goals of moral deliberation for themselves and their practice	Survey (Likert scale)	(1) to get knowledge of and insight in moral issues = 8.1 (2) to influence my attitude with respect to the case = 7.3 (3) to influence my behaviour with respect to the case = 7.1 (4) to improve my skills in dealing with moral issues = 7.5 (5) to deliver an answer or solution to the moral problem = 6.4 (6) to reach consensus within the group = 6.2 (7) to pay attention to reasons and arguments = 8.0 (8) to pay attention to feelings = 7.9 (9) to improve mutual understanding = 8.0 (10) to improve mutual cooperation = 7.9 (11) to active my job motivation = 9.7 (12) to frees my mind = 6.7 (13) to make me a better professional = 7.3 (14) to improve quality of care indirectly = 7.7 (15) to better ground decisions and reflect more on them = 7.7	[76]
Moral Distress	Address PICU provider moral distress	Survey (Pre/post survey using Moral Distress Scale Revised (MDS-R) (21-items) to rate "chronic" moral distress; every other month during data collection providers rated their "acute" moral distress using the expanded Moral Distress Thermometer (MDT)—single item scale with option to identify factors that contributed to moral distress)	There were three items on the instrument that showed statistically significant improvement in moral distress for nurses for both matched and aggregate data comparisons. On the aggregated comparison for nurses, four additional items showed a statistically significant drop in moral distress ‘‘Clinical Situations’’ represented the single most frequent contributing factor to moral distress	[89]
Experience and Impact of CEC	Overall assessment of CEC, impact on individual and values, and respondents’ expectations	Survey (11 items adapted from a tool developed by White, Dunn and Homer [47] and outcomes measures for EC (ASBH 2011)	Overall assessment of ECS was favourable. More than 90% felt the consultant explained things well, more than 80% felt the consultation validated the team's approach and provided support, and more than 70% felt the ECS clarified uncertainty, gave them a better understanding of ethical issues, and helped resolve a patient care problem. More than 80% felt the CEC recommendations were consistent with the organization's values, respected the respondent's values, and were consistent with their personal values. More than 60% felt the CEC helped clarify the values of the patient and/or patient's family, and helped respondents clarify their own values. Qualitative interviews uncovered some comments suggesting the EC could have communicated more effectively with members of the health care team	[90]
Experience of CES	Describing the experience of CES among professionals (“meeting in an ethical free zone”)	Qualitative interviews	CES sessions offered a chance to meet in an ethical free-zones allowing various professionals to relate to one another outside of roles, develop confidence to express points of view, and increase trust within the team. These "ethical free zones'' allowed them to develop a more integrated understanding, acquiring both knowledge and a more comprehensive view of it. The intervention seems to improve ability to act in practice, seeing CES as a way of becoming more prepared for dealing with care issues and developing resolutions from a shared standpoint	[54]
Experienced outcomes of CEC	The extent to which respondents had experienced outcomes (changes to perspective) during MCD or in their daily practice (developed skills, better managements, courage, security, greater awareness, etc.)	Survey (Euro MCD Instrument)	Percentage of respondents at T1 (not, somewhat, quite, and very): (1) develop skills to analyze ethical conflict = 3 (not), 31 (somewhat), 65 (quite and very) (2) more open communication = 4, 22 74 (3) consensus gained among co-workers re: situation management = 6, 36, 59 (4) enables better stress management = 22, 34, 45 (5) contributes to development of practice/policies = 12, 45, 43 (6) gives more courage to express ethical standpoint = 11, 30, 60 (7) feeling more secure to express doubts or uncertainty = 12, 30, 58 (8) better mutual understanding of other's reasoning = 3, 21, 77 (9) seeing the situation from different perspectives = 2, 19, 79 (10) more awareness of recurring situations = 7, 25, 68 (11) increase awareness of complexity of situation = 6, 26, 69 (12) enhancing understanding of ethical theory = 13, 37, 50 (13) enables decisions on concrete actions to manage situation = 9, 35, 55 (14) greater opportunity to have say = 6, 27, 67 (15) enhances possibility to share difficult emotions and thoughts = 4, 27, 70 (16) finding more courses of action = 5, 31, 64 (17) listening more seriously to other opinions = 12, 27, 61 (18) increase awareness of own emotions = 12, 31, 58 (19) strengthen self-confidence = 12, 32, 56 (20) develops ability to identify core ethical issues = 8, 35, 58 (21) more critical examination of existing policy/practice = 14, 34, 53 (22) more constructive management of disagreements = 14, 35, 52 (23) gaining more clarity about responsibility = 10, 34, 57 (24) enhancing mutual respect = 10, 28, 63 (25) more awareness of preconceived notions = 12, 31, 57 (26) better understanding of what it means to be a good professional = 12, 32, 56 Percentage of respondents at T2 (not, somewhat, quite and very): (1) develop skills to analyze ethical conflict = 2, 30, 68 (2) more open communication = 3, 21, 76 (3) consensus gained among co-workers re: situation management = 3, 36, 60 (4) enables better stress management = 13, 45, 43 (5) contributes to development of practice/policies = 9, 44, 47 (6) gives more courage to express ethical standpoint = 7, 24, 70 (7) feeling more secure to express doubts or uncertainty = 5, 27, 68 (8) better mutual understanding of other's reasoning = 1, 21, 78 (9) seeing the situation from different perspectives = 1, 23, 76 (10) more awareness of recurring situations = 1, 28, 71 (11) increase awareness of complexity of situation = 3, 22, 75 (12) enhancing understanding of ethical theory = 7, 36, 57 (13) enables decisions on concrete actions to manage situation = 4, 34, 62 (14) greater opportunity to have say = 2, 18, 80 (15) enhances possibility to share difficult emotions and thoughts = 2, 24, 74 (16) finding more courses of action = 2,30, 68 (17) listening more seriously to other opinions = 3, 18, 80 (18) increase awareness of own emotions = 5, 27, 67 (19) strengthen self-confidence = 7, 31, 62 (20) develops ability to identify core ethical issues = 4, 33, 63 (21) more critical examination of existing policy/practice = 8, 36, 56 (22) more constructive management of disagreements = 11, 33, 57 (23) gaining more clarity about responsibility = 4, 30, 66 (24) enhancing mutual respect = 4, 24, 72 (25) more awareness of preconceived notions = 5, 37, 58 (26) better understanding of what it means to be a good professional = 5, 36, 60	[59]
Learning Effects and Enhanced emotional support and Moral Reflexivity and Improved Moral Attitude	Becoming more aware of certain issues and moral dilemmas after they were discussed at a MCD and able to apply learning to similar cases This domain included 5 items: (1) Enhances possibility to share difficult emotions and thoughts with co-workers; (2) strengthens my self-confidence when managing ethically difficult situations; (3) Enables me to better manage the stress caused by ethically difficult situations; (4) Increases awareness of my own emotions regarding ethically difficult situations; (5) I feel more secure to express doubts or uncertainty regarding ethically difficult situations This domain includes 5 items: (1) Develops my skills to analyze ethically difficult situations; (2) increases my awareness of the complexity of ethically difficult situations; (3) develops my ability to identify the core ethical questions in the difficult situations; (4) I see the ethically difficult situations from different perspectives; (5) enhances my understanding of ethical theories (ethical principles, values, norms) This domain includes 5 items: (1) I become more aware of my preconceived notions; (2) I gain more clarity about my own responsibility in the ethically difficult situations; (3) I listen more seriously to others' opinions; (4) Gives me more courage to express my ethical standpoint; (5) I understand better what it means to be a good professional	Survey (Euro-MCD Survey)	Participants reported becoming more aware of certain issues and moral dilemmas after they were discussed at a MCD and being able to apply learning to similar cases Least often experienced outcomes during MCD session (t1, n = 22), assessed as "not experienced" or "experienced to some extent": "boosts my self-confidence when managing ethically difficult situations (67%)"; "enables me to better manage the stress caused by ethically difficult situations (63%)". Least often experienced outcomes in daily work after MCD sessions: "enables me to better manage the stress caused by ethically difficult situations (85%)." Least often experienced outcomes during MCD session (t1, n = 22): "enhances my understanding of ethical theories, principles, values and norms (65%). Most frequently experienced outcomes in daily work after MCD sessions (t1, n = 22): "I see ethically difficult situations from different perspectives (55%). Least often experienced outcomes in daily work after MCD sessions: "enhances my understanding of ethical theories (75%) Most frequently experienced outcomes experienced during MCD session (t1, n = 22): "my co-workers and I become more aware of recurring, ethically difficult situations (85%). Most frequently experienced outcomes in daily work after the MCD session: "my co-workers and I became more aware of recurring, ethically difficult situations (55%)"	[93]

Subthemes explored under the personal factors domain included perceived value and outcomes (n = 3), experience (n = 2), moral distress (n = 2), learning (n = 1), confidence (n = 1), and clarity (n = 1). With respect to perceived value and outcomes, understandings consisted of value for self and practice, impact on the individual, impact on values, changes to perspective during the session, changes to perspective in practice, and whether any outcomes were experienced at all during the session and/or in practice. Interestingly, other subthemes did not demonstrate as much variation.

Findings of the studies that examined the perceived value of CEC resulted in a variety of understandings of perceived values and outcomes with respect to ethics interventions. Respondents most frequently reported ethics interventions as valuable for enhancing their understanding and awareness of ethical issues (n = 4), developing confidence (n = 2), fostering open communication and expression of feelings (n = 2), improving mutual understanding and cooperation (n = 2), enabling and delivering solutions (n = 2), improving skills (n = 2), and achieving consensus (n = 2). Brännström et al. described the experience as encouraging stakeholders to meet in an ethical “free zone” in which the nature of the intervention created a safe and inclusive forum for stakeholders to express their viewpoints and to be heard in a space where they felt confident and trusted [54]. Wocial, Molnar, and Ott recommended effective communication with the health care team as an area for improvement [90].

Notably, most respondents in the two studies measuring moral distress reported a reduction in levels of distress following CEC intervention. Respondents in these studies consisted of health care providers, such as nurses and physicians. Moral distress levels were not measured among patients, families, or other stakeholder groups [66, 89]. Other studies reported perceived increases in clarity, confidence, and learning among providers [79].

#### Resource outcomes

A few studies reported on resource outcomes (n = 5). Data collection methods consisted primarily of medical record review. Studies coded under this domain evaluated outcomes in terms of service users’ consumption of health-related resources and cost or cost avoidance (Table 9).

**Table 9:** Resource factors reporting

Resource factors assessed (name construct)	Outcome description	Outcome measure	Results	Reference #
Length of Stay	Length of stay in hospital and length of stay in ICU	Record review (Chart review)	Membership in ethics proactive group and discharge status were significantly related to length of ICU stay** Those who died had predicted ICU stays of 7 days less than patients who survived; members in ethics proactive group had predicted ICU stays 6 days less than other 2 groups	[60]
Length of Stay	Number of inpatient hospital days	Record review (Medical record)	In patients who failed to survive to hospital discharge, intervention group had fewer days in hospital than control (split evenly between ICU/non-ICU hospital days** Intervention group had greater % of pts with 4–9 days and fewer pts with > 10 days, compared to control group (therefore ethics consultation more effective in reducing lengths of stay among those who would otherwise remain in hospital for 10 days or more)	[64]
Consumption of medical resources	Length of ICU stay, length of hospital stay, post-conflict length of ICU stay, post-conflict length of hospital stay (post-conflict = after occurrence of medical uncertainty or conflict regarding value-laden issues)	Record review (Medical record)	Participants in HCEC group showed significant reductions in entire ICU stay** and entire hospital stay* Participants in HCEC group had shorter ICU stay** and shorter hospital stay** after medical uncertainty/conflict than participants in UC group	[65]
Days in Hospital	Hospital length of stay and ICU length of stay	Record review (Medical record)	Hospital LOS (median days): Intervention (N = 56): N = 23 Control (N = 52): N = 21 ICU LOS (median days): Intervention (N = 56): N = 11 Control (N = 52) l: N = 11	[48]
Total cost of stay	Total cost of hospital stay	Record review (Medical record review subjected to cost assessment)	Intervention (N = 52): $167,350.00 Control (N = 56): $164, 670.00	[48]
Cost avoidance	Costs for patient's tx and expenses that were avoided or added for each patient who received a consultation Total Cost avoidance = hospitals' variable costs (direct patient care like supplies, nurses, technicians, etc.) + fixed costs (hospital overhead e.g., managers, administrators, equipment depreciation, etc.) Cost-Avoidance Factors: days in hospital, cost of resuscitation, surgical/diagnostic procedures, other, hospital expense for consultation service	Mixed methods (Medical records for actual financial hospital costs; interview with primary physician who indicated whether ethics consultation helped patient avoid medically inappropriate/undesired tx, whether consultation supported continuing tx/adding new tx, or no effect on patient's tx—this information was provided to the hospital comptroller who determined the costs for the patient’s treatment and expenses avoided)	Ethics consultation resulted in substantial cost avoidance: 20/29 consultations resulted in cost avoidance; 3/29 resulted in "potential cost savings," would've resulted in savings if primary physician accepted consultant's recommendation Hospital avoided $143,683 in variable costs and **$288,827 in total costs**; savings obtained by decreasing length of hospital stays, costs associated with resuscitation, # surgical/diagnostic procedures, other factors Hospital expenses for ethicist/supporting resources = **$12,000**	[75]
Hospital costs	Total inpatient stay costs were calculated as total service costs plus daily acute inpatient and ICU room-and-board costs; costs then aggregated to level of person for the hospital stay Estimated net cost of an ethics consultation practice from diff sources: annual cost of practice itself at ~ $150,000, estimated to be approx 1/2 FTE physician and full-time admin assistant plus office space in hospital including overhead; cost calculated as estimated incremental per person cost savings of intervention x # consultations expected in a year	Record review (Medicare cost reports obtained from finance departments of study hospitals)	6 hospitals averaged 40 ICU beds with ~ 50pts per year = savings of $5246 for reductions in nonbeneficial treatment among those who died in hospital = estimated savings of hospital tx costs $157,380 with an ethics consultation practice	[64]
Hospital charges	Charges for patients	Record review (Records obtained through fiscal services)	Proactive group patients who died had 16% reduction in average charges compared to baseline and 33% reduction compared with control patients; but was NOT statistically significant	[60]

All four studies that evaluated outcomes in terms of length of stay sought to examine whether length of stay was reduced following ethics intervention, both in terms of length of hospital stay (n = 4) and length of stay in the ICU, specifically (n = 3) pre- and post-intervention. While two studies denoted a shorter length of stay in the intervention arm compared to the control [64, 65], one study identified no statistically significant difference [48], and one study found length of stay in relation to discharge status significant [60].

Four studies reported on the subthemes cost or cost avoidance. These studies measured the financial impacts of CEC or used hospital records to compare cases involving ethics interventions to those that did not. Cost was described as total cost of stay, inpatient stay costs (total service cost + acute inpatient cost + ICU room and board), net cost of consultation, and charges for patients. Cost avoidance included variables such as cost for treatment, expenses avoided or added for patients receiving consultation, and total cost avoidance (variable costs + fixed costs) (Table 8).

Outcomes across studies reporting on cost and cost avoidance were not congruent with respect to actual and perceived impact of CEC. A study by Meltzer, Heilicser, and Siegler examining cost avoidance through retrospective record review drew a strong connection between ethics intervention and cost, reporting a $288,827.00 total cost avoidance over a six-month period, with savings obtained by decreasing length of stay, costs associated with resuscitation, number of surgical and diagnostic procedures, among other factors. This was compared to the expense of ethics support and resources, which was reported to be $12,000 for each patient who received consultation. Qualitative reporting in this study reflected similar outcomes: 69% of consultations resulted in cost avoidance, and an additional 10% resulted in *potential* cost savings. Researchers in this study asserted that cost savings would have been greater if ethical recommendations were followed [75]. Similarly, Gilmer et al. demonstrated comparable findings with an estimated annual savings of $157,380.00 related to ethics consultation practice [64]. On the other hand, while reporting a reduction in average charges compared to baseline groups, two studies did not report significant differences between intervention and control with ethics intervention [48, 60]. Dowdy, Robertson, and Bander specifically asserted that despite a reduction in costs, this difference was not statistically significant enough to demonstrate the efficacy or causal relationship between ethics consultation and cost reduction [60].

## Discussion

The current study is the first scoping review focused on outcome measures in CEC across healthcare settings. The 48 studies were highly heterogeneous and varied considerably with regard to format and processes of ethical intervention, the credentials of interventionist, the population of study, the outcomes reported, and the measures employed. In addition, few studies used validated measures. Regarding the quality domain, which was the most frequently reported domain, “usefulness” was the most common feature of quality and related to a variety of goals and processes of the CEC or was a standalone assessment of overall experience with CEC. Across the various studies that assessed usefulness within this domain, we did not identify consistency in characteristics of this subtheme, CEC approaches, population sampled, measures, or how potential biases about the outcome of the consultation (agreement/disagreement with the decision or recommendation) were managed in this context. Standardization of outcomes would be an important first step in helping to ensure reliable measurements and meaningful comparisons.

Previous systematic reviews have highlighted the methodological limitations of studies evaluating CEC effectiveness and the challenges associated with identifying relevant outcomes [7, 36]. To address these shortcomings, recent research has attempted to identify relevant outcomes using the Delphi method of consensus [95]. Although this study represents a strong starting point, it is not comprehensive and did not engage all relevant stakeholders. This is salient given the broad variety of persons that CEC serves, including patients. It is important to address patient-reported outcomes, as patient voices are increasingly recognized as central to research legitimacy and scientific advancement. Additionally, McClimans et al. focused more broadly on the role of support services in clinical ethics. However, without clear understanding of what these services encompass, and lack of standardized intervention, the field requires further research that addresses CEC specifically to more accurately evaluate and assess the effects of ethics interventions. As was the case with the McClimans et al. study, the current review notes the omission of patient perspectives in CEC evaluations. Most respondents in the studies reviewed were healthcare professionals, and very few surveyed the views of patients and families in assessing CECs.

The lack of consistent constructs, variation in how constructs are named and understood, different models of CEC intervention, and a lack of validated measurement tools detract from our ability to build an evidence base for CEC. Ultimately, without this evidence base, our ability to meaningfully support patient and professional decision-making remains in question as there is uncertainty regarding CEC effectiveness. Moreover, our ability to engage with what constitutes ‘good’ patient care may be compromised, especially within the context of an increasingly diverse patient population.

The findings of our review need to be interpreted within the broader socio-political context where more evidence is being shared regarding the colonial structures upon which our healthcare systems are built [96–98]. In particular, evidence pertaining to racist practices and policies, conscious and unconscious bias, and the outright discrimination and inequities in access to care experienced by racialized and marginalized groups [99, 100]. As a field, bioethics, and by extension those who practice CEC, are also impacted by conscious and unconscious bias and must grapple with how best to combat racism and other forms of oppression in the provision of our services and in health care more generally [101–103]. Historically, the field has responded to racism or issues related to racial oppression with a view towards ‘neutrality’ or the “idea that ideal [ethical] deliberation would ignore race and hence prevent bias” [104]. However, neutrality perpetuates racism by ignoring the systemic injustices experienced by racialized and marginalized groups that differentially affect their health. Neutrality is thus not only inappropriate for ensuring anti-discriminatory and anti-racist practices, but it is ultimately an affront to human dignity and the right to safe, accessible care. At present, we do not know whether CEC is doing a good job in addressing racism and promoting social justice, since this review reveals that CEC evaluation is not inclusive of diverse patient perspectives nor are we measuring outcomes such as anti-racism, patient safety and the provision of culturally appropriate care. Without a more inclusive understanding and evidence base for CEC, we risk grave human rights and safety implications for those who experience marginalization and oppression due to intersecting aspects of their identity, such as race, class, age, ability, and gender.

To resolve the lack of diverse perspectives and consensus in evaluating CEC, we offer two potential recommendations: enhanced international collaboration and the development of a core set of outcomes as identified in the MRC framework, both of which are to be guided by a commitment to the principles of equity, diversity and inclusion.

### International collaboration

Almost all the studies included in this scoping review were conducted in the United States (n = 27) or European countries (n = 18). Other countries were Japan (n = 1), Taiwan (n = 1), and Chile (n = 1). It may be advantageous for ethicists to collaborate on research internationally even though study sites and roles may be unique to different jurisdictions. In order to be more attentive to and inclusive of the perspectives of diverse groups globally, understanding of how CEC might differ in varied cultural contexts and the nuances of CEC beyond European and Westernized approaches to CEC may provide valuable learnings of benefit to all. There is also opportunity to seek insights and potential collaboration from colleagues involved in evaluating research ethics review and oversight (e.g., the Consortium to Advance Ethics Effective Research Ethics Oversight (AERO)). Although there are important differences between research ethics and CEC, the challenges with measurement and identifying relevant outcomes within a value-laden context are similar.

### Set of core outcomes

The current scoping review highlights the need to create a set of core outcomes through a comprehensive stakeholder engagement process that considers the action-guiding values of equity and anti-oppressive practices. The creation of this outcome set could also consider the agreement and disagreement among various stakeholders on the importance of each outcome, and note any limitations that should be recognized when applying these outcomes in empirical study. For example, the context and values-plurality of CEC has been recognized as an important factor in identifying outcomes in ethics consultations. Problems arise with pragmatic outcomes such as non-beneficial treatment or cost and satisfaction, when these overwhelm or conflict with the ultimate goals of CEC such as addressing value-related conflicts [11]. Defining non-beneficial treatment solely by measuring a patient’s days in the ICU could, after all, be seen as discordant with a fundamental goal of CEC, which is to ensure respect for patients’ values. The meaning of “benefit,” by most accepted accounts, is contingent on the patient’s wishes and values, which are to be understood as culturally and historically located, as well as dependent upon the unique circumstances of the case [11]. A patient’s desire to continue with ventilator support until their grandchild is born, for example, might strike the clinical team as a poor use of healthcare resources, but it is not inconceivable that a good CEC outcome would include the recommendation to retain the patient’s full code status until that wish is fulfilled. There may be an additional layer of cultural meaning ascribed to beginning of life rituals or religious practices that is contributing to the patient’s wishes, in which case supporting the recommendation to maintain full code status would also respect the patient’s cultural or religious identity.

A core outcome set can identify the most relevant outcomes based on the intervention (e.g., ethics committee or moral case deliberation), stakeholder perspectives and empirical research, and the limitations of these outcomes. This allows for flexibility in applying outcomes to a particular empirical study on CEC effectiveness while preserving the theoretical underpinnings of the particular CEC, context specificity of CEC, and the value-laden nature of the study outcome. Standardization, then, can (in time) be constructed within a robust and comprehensive core outcome set with accepted definitions and validated measures. Contemporaneously, the actual application of outcomes to the study of CEC will depend on the context and intervention. This requires multi-variable measurement to assess a thorough combination of relevant outcomes. Description of the methods and outcomes and any methodological limitations will need to be described in the study, but this is similar to any scientific enterprise and complex intervention where value-neutrality cannot be assumed. The process outcomes identified allow for flexibility in terms of CEC and values—thus they might be the preferred outcomes. Also, the outcomes identified may not be complete; there may be additional outcomes that have not yet been identified but that would be regarded as important by relevant stakeholders. Thus, pursuant to The Core Outcome Measurement in Effectiveness Trials initiative (an international collaborative that has described methodology to pursue core outcome sets) future research should seek to engage stakeholders and to develop consensus engagement and consultation.

### Limitations

This study was confined to peer-reviewed academic literature and did not retrieve grey literature that might have shed light on some of the issues discussed. Further, the study method did not analyze or categorize the set of articles in terms of publication year; results published in the last 5 years were treated in the same way as results published 20 years ago. Finally, although the search strategy was not limited to English language, one study that was potentially eligible for inclusion in the review based on its title was excluded at full-text screening because it was not written or translated into English.

## Conclusion

There is a need for the international clinical ethics community to determine standardized outcome measures for CEC evaluation research. Despite the benefits of standardized outcomes in research, there is also a need to resist the gravitational bias of evidence-based medicine and the hegemony of the physical sciences in the quest for definitive cause and effect [105]. The MRC framework, while on the one hand offers a useful guide to position CEC as relevant to health care, should be recognized as reproducing the development of complex interventions within a positivist paradigm inherent to medical practice and clinical science generally. There is a need to broaden the framework to include alternate epistemologies, including traditional and Aboriginal ways of knowing, and an anti-oppressive lens. Conflicts in values between patients and professionals, or between patients and families and clinical teams and hospital administrators, are not easily resolved and may relate to systemic injustices and historical trauma. These issues do not lend themselves to methodologies and methods that privilege the dominant perspective, observable facts, and concrete causality. The values-based realm of clinical ethics is context-bound, difficult to measure, and requires highly skilled facilitation to be effective. As is the case with other fields that encompass complex human behaviors and values (e.g., educational science), generalizable knowledge of CEC outcomes constitute one part of the overall research life-cycle. If the goal is to improve understanding of ethics in healthcare practice, producing generalizable knowledge should not necessarily be viewed as “the pinnacle and primary goal of research activity” [105], although it can provide an important contribution. While we appreciate the challenges to precision in the development of measures for CEC, we should not rest content with less precision than our field will allow. As Aristotle’s famous aphorism from the *Nichomachean Ethics* concludes:

Our discussion will be adequate if it has as much clearness as the subject-matter admits of, for precision is not to be sought for alike in all discussions, any more than in all the products of the craft [106].

## Data Availability

The datasets analysed during the current study are available from the corresponding author on reasonable request.

## Footnotes

1 We acknowledge the problematic use of the term ‘stakeholder’ to identify groups who have an interest in CEC or whose lives or well-being will be impacted by CEC. This term has its origins in business and corporate relations literature, and has been identified by Indigenous and Aboriginal peoples as not respectful of their relation to their own traditional land. Indigenous and Aboriginal peoples are not stakeholders in their own lands, but have roles in governing, protecting and stewarding the territories for their members and future generations. See, for example, pg. 24 of https://www.iphcc.ca/wp-content/uploads/2020/02/Cultural-Competency-Guideline-Report-June-2018.pdf. Henceforth, we use ‘stakeholder’, a term that is ubiquitous in the engagement literature, with this deeper awareness and in consideration of diverse communities.

2 Quality improvement studies are quasi-experimental and frequently involve pre- and/or post-test study designs to evaluate the effectiveness of CEC.

3 This includes quality studies that examine healthcare professionals as a standalone group, or in conjunction with other study populations (e.g. healthcare professionals and families).
